# Phytotoxic Effects and Mechanism of Action of Essential Oils and Terpenoids

**DOI:** 10.3390/plants9111571

**Published:** 2020-11-13

**Authors:** Mercedes Verdeguer, Adela M. Sánchez-Moreiras, Fabrizio Araniti

**Affiliations:** 1 Mediterranean Agroforestry Institute (IAM), Universitat Politècnica de València, Camino de Vera s/n, 46022 Valencia, Spain; merversa@doctor.upv.es; 2 Department of Plant Biology and Soil Science, Faculty of Biology, Universidade de Vigo, Campus Lagoas-Marcosende s/n, 36310 Vigo, Spain; 3 CITACA, Agri-Food Research and Transfer Cluster, Campus da Auga, University of Vigo, 32004 Ourense, Spain; 4 Department AGRARIA, University Mediterranea of Reggio Calabria, Loc. Feo di Vito, 89100 Reggio Calabria, Italy; fabrizio.araniti@unirc.it

**Keywords:** essential oils, terpenoids, phytotoxicity, mode of action, natural herbicides, secondary metabolites, weed control

## Abstract

Weeds are one of the major constraints in crop production affecting both yield and quality. The excessive and exclusive use of synthetic herbicides for their management is increasing the development of herbicide-resistant weeds and is provoking risks for the environment and human health. Therefore, the development of new herbicides with multitarget-site activity, new modes of action and low impact on the environment and health are badly needed. The study of plant–plant interactions through the release of secondary metabolites could be a starting point for the identification of new molecules with herbicidal activity. Essential oils (EOs) and their components, mainly terpenoids, as pure natural compounds or in mixtures, because of their structural diversity and strong phytotoxic activity, could be good candidates for the development of new bioherbicides or could serve as a basis for the development of new natural-like low impact synthetic herbicides. EOs and terpenoids have been largely studied for their phytotoxicity and several evidences on their modes of action have been highlighted in the last decades through the use of integrated approaches. The review is focused on the knowledge concerning the phytotoxicity of these molecules, their putative target, as well as their potential mode of action.

## 1. Introduction

Plants are sessile organisms daily exposed to various biotic and abiotic stress factors and continuously involved in competition with other organisms for edaphic resources. Such necessity to cope with stress and to fight for species survival has pushed these species in evolving defense mechanisms and in increasing the competitive capacity in favor of the single plant or the whole species [1,2]. 

The only strategy that could be adopted by plants to face these challenges consists in adjusting their physiological state in preparation for and/or in response to these threats in order to improve their well-being and survival [1]. In addition to physiological adjustments, plants have evolved the production of secondary metabolites, not necessary for cell survival but pivotal for the survival of the species, mainly involved in plant–organism communication (bacteria, plants, insects, fungi etc.) and often used as chemical weapons, capable of positively/negatively affecting the growth and development of neighboring species [3].

Secondary metabolites such as phenolic compounds, short chain fatty acids, terpenoids, alkaloids among others, can be constantly produced by the plants or their production could be induced ex novo, since it has an energetic cost for the plant, by several factors such as both biotic and abiotic stresses, kin recognition, climatic changes and phenological stages [3].

A both constitutive and inducible defense system adopted by plants to communicate, compete and cope with stress is the production of volatile organic compounds, VOCs, chemicals that are involved in several plant processes such as (i) attracting beneficial insects and pollinators, (ii) protecting plants from heat, cold and elevated ozone concentration, (iii) defending plants from herbivory and (iv) priming neighboring species against biotic and abiotic stresses [4,5].

Moreover, these chemicals are involved in most complex trophic interactions playing a role of semiochemicals, which highlight the wide specialization of these compounds. For example, a recent detailed review was focused on the microorganisms–plants–insects tritrophic interaction. Authors have been able to document that microorganism-induced volatile communication could significantly influence insect behavior [6]. VOCs are synthesized, accumulated and then secreted by specialized tissues or cell types and then stored in complex and specialized secretary structures, generally classified into three types: superficial glandular trichomes (Lamiaceae and Asteraceae, e.g., mint, sage and chamomile); internal resin ducts, common to conifers; and embedded secretory cavities, characteristic of *Eucalyptus* and *Citrus* (schizogenous origin in Myrtaceae and schizolysigenous in Rutaceae). All structural types contain specialized biosynthetic cells. Additionally, in some cases, essential oils (EOs) are stored in undifferentiated cells, like in Lauraceae [7,8]. From the chemical point of view, VOCs belong to two groups (i) terpenes and terpenoids (a terpene containing oxygen) and (ii) aromatic and aliphatic constituents. Regardless, terpenoids are the most representative and abundant compounds released by plants.

Terpenes are the unsaturated hydrocarbons, which have a distinct architectural and chemical relation to the simple isoprene molecule. The simplest terpenes are monoterpenes (molecular formula C_10_H_16_), which are biosynthesized through the head to tail union of two isoprene units [9]. The general formula used to express their composition is (C_5_H_8_)_n_. Depending on “*n*” number we can have monoterpenes (*n* = 2), sesquiterpenes (*n* = 3), diterpenes (*n* = 4) etc. [9]. Terpenoids are terpenes that, through an enzymatic-driven biochemical modification, lose a methyl group, which is substituted by oxygen addition [9].

Terpenoids biosynthesis is localized in both cytoplasm and plastids of plant cells via the methyl-d-erythritol-4-phosphate pathway (MEP), which provides the precursors for the biosynthesis of the volatile hemiterpenes (C5), monoterpenes (C10) and diterpenes (C20), via the mevalonic acid pathway (MAV), from which originate the volatile sesquiterpenes (C15) [9,10] and via the shikimic acid pathway, which heads to phenylpropenes [11].

Generally, VOCs are constituted by few major components at relatively high concentrations (20–70%) and several components that are present in trace amounts. It was generally assumed that the biological properties of VOCs were mainly determined by their major components. However, relatively recent studies have also demonstrated that trace elements can be determinant in plant defense strategies and could play a pivotal role by acting synergistically in improving the biological activity of the major constituents [12].

In the last few years, because of their complex chemical composition, their high biological activity and being safe compounds for the environment and human health, VOCs gained a renewed interest in several industrial areas, which pushed both researchers and industries in finding strategies to extract and concentrate them. VOCs extraction and concentration are generally achieved by hydrodistillation and/or mechanical means (e.g., cold-press extraction) and the final product are essential oils (EOs) or aetherolea.

EOs are natural, volatile, complex mixtures of compounds, consisting in aromatic liquids, obtained from different plant material, like flowers, roots, bark, leaves, seeds, peel, fruits, wood or the whole plant [7,13,14]. They are defined by the International Organization for Standardization (ISO, 2014) [15] as “a product obtained from natural raw material of plant origin, by steam distillation, by mechanical processes from the epicarp of citrus fruits, or by dry distillation, after separation of the aqueous phase by physical processes.” This definition is common with other prestigious institutions, such as the European Pharmacopoeia (Ph. Eur.) [16], or the Association Française de Normalisation (AFNOR). EOs have great potential in agriculture for crop protection, as they possess antimicrobial [17] and antibacterial [18] properties, as well as insecticidal [19] and herbicidal activity [20,21].

The EO composition, which includes mainly lipophilic and highly volatile, scarcely water-soluble compounds, determines the properties and the biological activities of EOs [13,22,23,24]. The main compounds of EOs are terpenoids, principally mono- and sesquiterpenes, but also diterpenes can be found, all of them in the form of hydrocarbons, alcohols, aldehydes, ketones, ethers, esters, peroxides and phenols. Aromatic compounds are less frequent than terpenes but are characteristic of some EOs (e.g., eugenol is the main compound of clove EO (*Zygium aromaticum* (L.) Merr. et L.M.Perry), and *trans*-cinnamic aldehyde is the major constituent of cinnamon EO (*Cinnamomum verum* J.Presl). Aliphatic compounds (hydrocarbons, alcohols, acids, aldehydes, esters and lactones) can also be present in the EO composition [7,13,22,24]. Furthermore, other substances, such as fats, coumarins, anthraquinones and certain alkaloids, which are distillable, have been identified in EOs obtained by distillation. Some compounds are derived from glycosides, which are transformed during the distillation process [7]. In EOs from *Citrus* species, volatile and semivolatile compounds represent 85–99% of the EO composition, with the most frequent compounds being hydrocarbon and derived mono- and sesquiterpenes, followed by aliphatic and olefinic C6–C12 nonterpene aldehydes, alcohols, ketones, esters and acids, together with several aromatic compounds. The nonvolatile residue is mainly composed of flavonoids, coumarins, diterpenoids, sterols and fatty acids [25].

The qualitative and quantitative composition of EOs determines their quality, value and price on the market and it is not standard. It is necessary to know the causes of variability in EO composition to control and manage them [26], which can be divided in abiotic and biotic factors. Abiotic factors include growing conditions of the plant from which they are extracted, like climatic conditions (temperature, rainfall, humidity, light intensity, wind), soil conditions, agronomical practices (water supply, fertilization) and harvesting time [24,26]. The biotic/biological factors are the genetic/biological differences of the source plants and root colonization by symbiotic microorganisms. For example, differences in the EO composition can be found depending on the organ from which the EO is extracted. EO composition in some species is very stable but in others can have great variations, and different chemotypes can be found [24,26].

Approximately, 3000 different EOs have been described, and 300 of them are commercially important for their applications in the pharmaceutical, agronomic, food, sanitary, cosmetic and perfume industries [7,13].

The main botanical families that produce EOs are, according to Vigan [27] and Raut and Karuppayil [28], the Abietaceae, which contains *Pinus pinaster* Aiton, from which turpentine is obtained; the Cupressaceae, including thuja (*Thuja* spp.), cypress (*Cupressus* spp.) and juniper (*Juniperus* spp.); the Lamiaceae, one of the most important, which comprises basil (*Ocimum basilicum* L.), true or hybrid lavender (*Lavandula* spp.), marjoram (*Origanum majorana* L.), lemon balm (*Melissa officinalis* L.), mint (*Mentha* spp.), oregano (*Origanum* spp.), patchouli (*Pogostemon cablin* (Blanco) Benth.), rosemary (*Rosmarinus officinalis* L.) and sage (*Salvia officinalis* L.); the Myrtaceae, containing *Eucalyptus* spp., clove tree (*S. aromaticum*), myrtle (*Myrtus* spp.) and niaouli (*Melaleuca quinquenervia* (Cav.) S.T.Blake); the Lauraceae, covering cinnamon (*C. verum*), laurel (*Laurus nobilis* L.), rosewood (*Aniba rosaeodora* Ducke), clove nutmeg (*Pimenta dioica* (L.) Merr.) and sassafras (*Sassafras albidum* (Nutt.) Nees); the Rutaceae, which is another of the most important families, including a great number of EOs from *Citrus* fruits, that are the most popular natural EOs and account for the largest proportion of commercial natural flavors and fragrances [29], as lemon (*Citrus limon* (L.) Osbeck), lime (*Citrus aurantiifolia* (Christm.) Swingle), mandarin (*Citrus reticulata* Blanco), sweet and bitter orange (*Citrus sinensis* (L.) Osbeck and *Citrus* × *aurantium* L.) and grapefruit (*Citrus paradisi* Macfad.); the Ericaceae, which contains wintergreen (*Gaultheria procumbens* L.) and Labrador tea (*Ledum palustre* subsp. *groenlandicum* (Oeder) Hultén); the Asteraceae, which includes camomile (*Matricaria chamomilla* L.), tarragon (*Artemisia dracunculus* L.), sweet inula (*Dittrichia graveolens* (L.) Greuter) and gray santolina (*Santolina chamaecyparissus* L.); the Alliaceae, from which *Allium* genera have been the most studied [30,31]; the Apiaceae, which EOs have shown insecticidal properties (*Anethum graveolens* L., *Cuminum cyminum* L., *Foeniculum vulgare* Mill., *Petrosellinum crispum* (Mill.) Fuss) [32], and antioxidant and hepatoprotective potential (*Coriandrum sativum* L. and *Carum carvi* L.) [33]; the Poaceae, with lemon grass (*Cymbopogon citratus* (DC.) Stapf) being the most important representative; the Rosaceae, containing rose (*Rosa* spp.), which EO has been widely investigated [34,35]; Geraniaceae, which contains *Pelargonium* spp.; and Santalaceae with *Santalum* spp.

The use of EOs is continually increasing due to the strong demand of pure natural ingredients in many sectors [29]. In the food industry, EOs could be also used as natural antimicrobials for food preservation [14]. In the latest years in Europe, a great number of EOs has been approved for their use in agriculture, especially as biocides, such as *Mentha arvensis* L. and *Mentha spicata* L., *Artemisia alba* Turra and *Citrus* × *aurantium* L., among others [24]. There are commercial products available, based on EOs, used as fungicides or bactericides (e.g., BIOXEDA, from Xeda International, France, clove oil), as growth regulators (e.g., BIOX-M, from Xeda International, France, *Mentha spicata* EO) and as fungicides and insecticides (LIMOCIDE, from VIVAGRO SARL, France; OROCIDE, from Idai Nature, Spain; and PREV-AM, from ORO AGRI INTERNATIONAL LTD, all three sweet orange EO) [24] but there are still not commercial herbicides based on EOs available in the European market, although there is a European patent involving the use of EOs to control weeds [36]. In the USA market, there are many commercial herbicides formulated with EOs readily available, which will be reviewed in detail in Section 5.

## 2. Role of Terpenoids in Plant–Plant Interactions

The understanding that EOs and/or their constituents could be a source for the production of new formulations employable in weed management is strictly connected with ecological studies focused on plant–plant interactions. In fact, several botanists and ecologists have been able to demonstrate that plants, through the release of volatile organic compounds (VOCs), are able to alter the growth of neighboring species affecting the composition of plant communities in natural ecosystems [37,38,39].

Through the release of VOCs, mainly terpenoids, plants might induce changes to the neighbor’s phenotype [40] or prime them, affecting the competitive interactions and defensive strategies under stress conditions [41]. Recently, Landi et al. [41] reported that salinity stress altered VOC profile in emitter sweet basil plants and those airborne signals promoted the earlier flowering of kin receivers, thus increasing their reproductive success.

Plant communities are characterized by a high density of plants that might interact and/or compete with genetically related neighbors, such as their offspring, and other species. During these interactions, plants are able to communicate with neighbors of different identity and/or under different conditions, through the activation of a huge variety of signals and responses. For example, as reported by Kegge et al. [42], differences in the ratio between red and far-red light conditions modulate the emission of VOCs released from barley, leading to an alteration of biomass allocation in neighboring plants. Similarly, Ninkovic [43] demonstrated using kin species that aerial plant–plant communication significantly affects biomass allocation in individual plants without altering the total biomass.

Although several evidences have been reported concerning the role of terpenoids in plant–insect and/or in plant–plant defense-related communication, only few evidences have been published concerning the ability of plants to inhibit and/or stimulate the growth and development of neighboring species. In fact, the majority of the experiments focused on this topic, aimed at describing this phenomenon, were carried out in vitro using pure compounds or concentrated VOCs (mainly essential oils) extracted by the donor species and not in natural ecosystems and/or in systems aimed to mimic field conditions. Anyway, few researches have tried to fill this gap and robust results have been published.

In 1964, Muller and Muller [44] demonstrated that the inhibition of growth of annual grassland species growing close to *Salvia leucophylla* Greene colonies was mainly due to camphor and cineole, two volatiles actively released by this aromatic species. They also reported that those volatiles, mainly terpenoids, were able to inhibit the growth of several soil bacteria influencing the spacing and patterning of grassland species [45,46]. Moreover, they demonstrated [47] that the soil collected under *S. leucophylla* bushes was characterized by a high phytotoxicity mainly due to the ability of dry soil colloids to adsorb terpenoids from the atmosphere.

These data were further confirmed by Nishida et al. [48], which demonstrated that monoterpenoids released by this species were characterized by an inhibitory activity on cell proliferation and DNA synthesis in the root meristem of *Brassica rapa campestris* L.

All these data suggest that a plant species could affect plant communities through the release of VOCs, acting on a multiscale level. In this context, Karban [49] reported that VOCs emitted by *Artemisia tridentata* Nutt. damaged leaves induced a significant inhibitory effect on the germination of neighboring unrelated species. Interestingly, the germination of sagebrush seeds was not affected at all by these volatiles, suggesting a strategy aimed at increasing the competitiveness of the species and modeling the plant community structure. Similarly, Araniti et al. [37] demonstrated that the VOCs released by *Dittrichia viscosa* (L.) Greuter subsp. *viscosa*, a pioneer Mediterranean shrub forming large monospecific communities, were able to affect the growth of the sensitive species *Lactuca sativa* L. altering its primary metabolism, inducing ROS (reactive oxygen species) burst and physical damages to the photosynthetic machinery.

Ninkovic et al. [50,51] reviewed the role of volatiles in plant competition and in tritrophic interactions, concluding that signals induced by VOCs could provide pivotal information concerning the genetic identity as well as the physiological status of the emitter. Such information can be further used to detect competitive neighbors and to activate, even before that competition takes place, competitive responses aimed at initiating specific growth responses that could increase their competitive capacity.

## 3. Herbicidal Activity of Essential Oils

The phytotoxic and herbicidal potential of EOs against weeds has been widely studied for their use as an alternative to synthetic herbicides. Since their discovery and development in the 1940s, synthetic herbicides have been the main method used for weed management. Their overuse has promoted the evolution of herbicide-resistant weed biotypes [52], as well as harmful effects to human and animal health [53] and the environment [54].

In the latest years, the research regarding EO application in pest management has increased greatly, due to the changes in the regulation of the pesticides market in the European Union (EU) (Directive 2009/128/EC). These changes are focused in achieving the sustainable use of pesticides, and implementing, as mandatory in the EU, the principles of integrated pest management (IPM), which gives priority to the use of nonsynthetic pesticides for weed control.

In this section, the studies about phytotoxic and herbicidal activity of EOs on weeds and crops are reviewed, reporting the research carried out during the latest twenty years. As there are many works that study the herbicidal activity of EOs from different *Eucalyptus* species, they will be analyzed separately. Some previous works also summarized the herbicidal activity of EOs [22,24,55,56], giving other interesting and complementary points of view.

The basis for the use of EOs in weed control is because they contain allelochemical compounds, mainly terpenoids, which can prevent the germination and growth of weed species [57,58]. In Table 1, the most important works regarding the herbicidal activity of EOs are summarized and ordered chronologically, focusing on the compounds that are involved and the species on which they were tested and the effects they promoted.

One of the first studies, which approached the investigation of the allelopathic compounds contained in the EOs composition from a practical point of view and considering their possible applications in agriculture to control weeds, was the work of Dudai et al. [57] (Table 1). They verified that EOs showed different potential to prevent weed seed germination and growth depending on the EO composition and on the species against which they were applied. Their results opened many opportunities to employ EOs for weed management, using different EOs depending on the weeds to be controlled. Other remarkable work is that of Tworkoski [20], as he tested the EOs in vivo under greenhouse conditions, when the majority of studies about EO herbicidal activity are carried out in vitro conditions. The reason to carry out in vitro assays with EOs is that in vivo assays confront some difficulties, like the quantity of EOs needed for the assays, or the proper formulation of the EOs to be mixed with water and to enhance their properties, as their persistence and penetrability. In Tworkoski’s work, many EOs (25) were tested and the main compound (eugenol) of the most active EO (*S. aromaticum*) was determined and it was verified that the herbicidal activity of *S. aromaticum* EO was due to this compound. Angelini et al. [58] tested different EOs (from *Rosmarinus officinalis* L., *Thymus vulgaris* L. and *Satureja montana* L.) and their main compounds on different weeds and crops (Table 1), finding that *S. montana* EO with 57% carvacrol was the most effective, completely inhibiting the germination of crops and weeds. It is noteworthy the work of Vokou et al. [59], who tested the allelopathic potential of 47 monoterpenoids of different chemical groups against *Lactuca sativa* L. germination and growth, determined that hydrocarbons, except (+)-3-carene, were the least inhibitory and acetates were the less inhibitory of oxygenated compounds. Whenever the free hydroxyl group of an alcohol turned into a carboxyl group, the activity of the resulting ester was considerably lower (against both germination and seedling growth). They found more active compounds effective against seedling growth (24 compounds) than against seed germination (only 5 compounds). The most active compounds, which controlled both processes, belonged to four groups of ketones and alcohols: terpinen-4-ol, dihydrocarvone and two carvone stereoisomers. In this research, the monoterpenes were also tested in pairs, and in half the cases they acted as expected by the activity shown individually, but in the other cases, antagonistic and synergistic interactions were detected. Thus, to predict the herbicidal activity of an EO based on its composition when it has many components is not easy, as the presence of minor compounds can alter the expected behavior of the main components of EOs.

Armirante et al. [60] investigated the herbicidal potential of EOs from aromatic plants Hyssopus officinalis L., Lavandula angustifolia Miller, Majorana hortensis L., Melissa officinalis L., Ocimum basilicum L., Origanum vulgare L., Salvia officinalis L., and Thymus vulgaris L. on Raphanus sativus L., Lactuca sativa L. and Lepidium sativum L. (Table 1) concluding that the EOs tested showed a good inhibitory activity against the germination and the radical length of the species assayed, dependent of the doses applied, and also the inhibitory activity increased with the total monoterpene content of the EO. They affirmed that the overall effect of EOs from aromatic plants cannot be predicted, unless the composition and the interactions between their constituents are known. These studies were continued with the work of De Almeida et al. [61], who tested the previously mentioned EOs together with EOs from species of Verbenaceae and Apiaceae families (Table 1). All the EOs assayed were active, inhibiting the germination and radicle growth of the three species tested, but the activity depended on the EO applied, the doses and the species against which they acted. The EOs with the greater herbicidal potential were those from T. vulgaris, M. officinalis, V. officinalis and C. carvi.

Campiglia et al. [62] tested in vivo, under greenhouse conditions, the herbicidal activity of cinnamon (*Cinnamomum zeylanicum* L.), lavender (*Lavandula* spp.) and peppermint (*Mentha* × *piperita* L.) EOs against *Amarantus retroflexus* L., *Sinapis arvensis* L. and *Lolium* spp. (Table 1). EO application reduced seed germination of all tested weeds. The most effective was *C. zeylanicum* EO, and the dicotyledonous weeds were more susceptible than the monocotyledonous, with *A. retroflexus* the most sensitive species. This research was continued by Cavaliere and Caporali [63], who tested the same EOs on seven weeds (*A. retroflexus*, *Solanum nigrum* L., *Portulaca oleracea* L., *Chenopodium album* L., *S. arvensis*, *Lolium* spp. and *Vicia sativa* L.) in vitro and in vivo, under greenhouse conditions (Table 1). All the EOs inhibited weed seed germination in the in vitro assays, and the dose to reach 100% inhibition depended on the EO and the weed species tested. The most effective EO was *C. zeylanicum*, and the most sensitive weeds were *A. retroflexus*, *P. oleracea* and *V. sativa*, which germination was completely inhibited by all EOs at 1.8 mg/L concentration, while *S. arvensis* and *Lolium* spp. were the most resistant weeds, as their germination was completely controlled only at the highest concentration (5.4 mg/L). The weeds showed different responses to the phytotoxic effects of EOs. They observed that the concentration of EOs had a greater effect on weed susceptibility than the type of EO used. However, in vivo assays showed that *A. retroflexus* was the most sensitive weed while *Lolium* spp. was the most resistant. *C. zeylanicum* EO was also the most active EO in vivo conditions (Table 1).

The bioherbicidal potential of *Artemisia scoparia* Waldst et Kitam EO was verified in vitro and in vivo on the weed species *Achyranthes aspera* L., *Cassia occidentalis* L., *Parthenium hysterophorus* L., *Echinochloa crus-galli* (L.) P. Beauv and *Ageratum conyzoides* (L.) L. [64] (Table 1). This work is very interesting because they also studied and described the physiological effects caused by the herbicidal activity of the EO on the weed species (Table 1).

The EOs from two species from Chile, *Peumus boldus* Molina and *Drimis winteri* J.R.Forst. et G.Forst. were tested with *P. boldus*, showing good herbicidal activity [24] (Table 1). The study of this EO continued in the work of Blázquez and Carbó [65], who tested *P. boldus* and *C. limon* EOs on *P. oleracea*, in Petri dishes with filter paper or filled with different types of soil (clay, silty clay, loam and sandy clay loam) and with sand. They confirmed that the EO activity depended on soil characteristics. At the highest dose (1 μL/mL) *P. boldus* EO completely inhibited *P. oleracea* germination in filter paper, sand and in clay and silty clay soils, being also very effective in loam soil. At low doses (0.250 μL/mL), it only showed significant effect in soils with clay and sand textures, with the lowest concentration applied (0.125 μL/mL) effective in soils with more clay content. *C. lemon* EO was not active at the doses tested.

*C. ladanifer* EO was tested in vitro against *Amaranthus hybridus* L., *P. oleracea*, *C. album*, *Erigeron canadensis* L. and *Parietaria judaica* L., completely inhibiting *A. hybridus* germination, and nearly blocking *E. canadensis* and *P. judaica* germination at all concentrations assayed. In *P. oleracea*, the EO was active only at the higher doses tested. *C. album* was the most resistant weed, the EO did not control its germination. Although inhibiting seed germination, the EO showed a selective behavior; it had strong phytotoxic activity by reducing seedling length, being effective in all species at all concentrations [66] (Table 1).

The work of Hazrati et al. [67] is remarkable, who tested a nanoemulsion (NE) of *Satureja hortensis* L. EO against *A. retroflexus* and *C. album*, in vitro and in vivo conditions, demonstrating the strong herbicidal potential of *S. hortensis* EO NE and the possibility to use it as a natural herbicide (Table 1). In another work, Hazrati et al. [68] also investigated the herbicidal activity of *R. officinalis*, *S. hortensis* and *Laurus nobilis* L. EO, and mixes of *R. officinalis* and *L. nobilis* EOs against one monocotyledonous weed (*Bromus tectorum* L.), one dicotyledonous weed (*A. retroflexus*) and one crop (*Solanum lycopersicum* L., tomato). These assays were carried out in vitro conditions only. The EOs and their mixtures strongly inhibited the germination and seedling growth of the species tested, with *A. retroflexus* the most sensitive.

Recently, the EOs from three *Copaifera* species (Leguminosae): *C. duckei* Dwyer, *C. martii* Hayne and *C. reticulata* Ducke (Leguminosae) from Amazon (Brazil), were tested for their herbicidal properties against two invasive plants native to the Brazilian Amazon, *Mimosa pudica* L. and *Senna obtusifolia* (L.) Irwin et Barneby, both belonging to Leguminosae family (Table 1). This work demonstrated the different herbicidal potential of EOs coming from different organs of the same plant, as the EOs obtained from the stems showed greatest inhibitory potential of the germination than those from the leaves, but it was very low (17.3% for *M. pudica* and 18% for *S. obtusifolia).* However, on root development, the EOs from leaves showed greater inhibitory potential than those from the stems, with values above 42%. The composition of EOs from leaves and stem were different; EOs from leaves had more constituents, especially in *C. martii*. This was the cause for their different activity. All EOs tested showed high inhibitory effects on *M. pudica* hypocotyl development, with values above 69%, with *C. reticulata* the most active (76% inhibition). *S. obtusifolia* was more resistant, the highest inhibition of hypocotyl development on this species was registered for *C. copaifera* EO (47.2%).

The EOs from *Thymbra capitata* (L.) Cav., *Mentha* × *piperita* L., *Santolina chamaecyparissus* L. and *Eucalyptus camaldulensis* Dehnh. were tested in vivo, in greenhouse experiments, applied in pre- and post-emergence (by watering and spraying), to control *Erigeron bonariensis* L. [69] (Table 1), one important cosmopolite weed, which affects many crops and has developed resistant biotypes to glyphosate, the worldwide most used herbicide [70]. Results showed that the method of application also determines the herbicidal potential observed. In pre-emergence assays, *T. capitata* EO was the most potent. In post-emergence assays, *T. capitata* EO was the most effective to control *E. bonariensis*, in both modes of application, irrigation and watering, but it showed a more rapid action when sprayed*. E. camaldulensis* was the second most active EO when applied by watering. *M. piperita* applied by spraying showed an efficacy similar to *E. camaldulensis* applied by watering and to *T. capitata*. *S. chamaecyparissus* was the least active EO. *T. capitata*, *E. camaldulensis* and *M. piperita* showed good herbicidal activity controlling this weed, and could be used as basis for the development of natural herbicides [69]. The study of the herbicidal potential of these EOs was continued, and also their effect on soil microorganisms was evaluated [71], verifying that only *T. capitata* EO when applied at the highest dose did not permit soil microorganisms to recover their initial functionality. *T. capitata*, *M.* × *piperita* and *S. chamaecyparissus* EOs were tested in vivo by irrigation against important weeds in Mediterranean crops, as the dicotyledonous *A. retroflexus* and *P. oleracea*, and the monocotyledonous *Avena fatua* L. and *E. crus-galli*. *T. capita* was the most effective EO, killing all weeds at the highest dose applied (12 µL/mL), except *P. oleracea*, which was eliminated in 90%. As *T. capitata* EO demonstrated the highest herbicidal potential, it was studied more in detail, through in vitro and in vivo experiments carried out on many weeds [72] (Table 1). In vitro experiments showed the strong herbicidal potential of *T. capitata* EO, which controlled completely the germination of different common and problematic weed species, although they showed different sensitivity to the EO, as it blocked the germination and seedling development of *E. canadensis*, *Sonchus oleraceus* (L.) L. and *C. album* at 0.125 μL/mL (more sensitive species), of *Setaria verticillata* (L.) P. Beauv., *A. fatua* and *Solanum nigrum* L. at 0.5 μL/mL, of *A. retroflexus* at 1 μL/mL and of *P. oleracea* and *E. crus-galli* at 2 μL/mL (more resistant species). In greenhouse experiments, *T. capitata* EO was tested in pre- and post-emergence by irrigation against the weeds present in a citrus orchard soil seedbank. It showed strong herbicidal activity at 4 μL/mL. In vivo experiments under greenhouse conditions were carried out to test *T. capitata* EO, applied by spraying in post-emergence on *P. oleracea*, *A. fatua* and *E. crus-galli* plantlets. The species showed different sensibility to the EO, with *E. crus-galli* the most resistant. With the objective to determine which mode of application was more effective, spraying or watering, *T. capitata* EO was applied on *A. fatua* by spraying and by irrigation. Comparing the data obtained in *A. fatua* and in other species, it was concluded that *T. capitata* EO was more effective at the same doses applied by irrigation in monocotyledons and by spraying in dicotyledons [72].

Finally, we will summarize the works about the herbicidal activity of EOs from different *Eucalyptus* species. The strong herbicidal potential of *Eucalyptus* EOs has been demonstrated [73,74,75]. One of the most studied EOs from *Eucalyptus* species is the EO from *E. citriodora* Hook (the accepted name of these species is *Corymbia citriodora* (Hook.) K.D.Hill et L.A.S.Johnson), which has been tested against different weed species in many works [75,76,77] (Table 1), concluding that it can be an excellent candidate to be used as bioherbicide. However, Ibáñez and Blázquez [78] tested *E. citriodora* EO against *P. oleracea*, *E. crus-galli* and *Lolium multiflorum* Lam in vitro conditions finding no significant inhibitory effects on seed germination and hypocotyl length on the tested weeds at the concentrations assayed (0.125–1 µL/mL) (Table 1).

Verdeguer et al. [73] tested the herbicidal potential of *E. camaldulensis* EO rich in spathulenol (41.46 ± 3.04%), showing high herbicidal potential against *A. hybridus* and *P. oleracea*. (Table 1)

The herbicidal potential of *Eucalyptus tereticornis* Sm EO was tested against *E. crus-galli* [79] (Table 1) demonstrating great possibilities to be used as bioherbicide to control this noxious weed. The EO from *E. citriodora* showed good potential as bioherbicide, as it controlled in vitro and in vivo *S. arvensis, S. oleraceus, Xanthium strumarium* L. and *A. fatua* [77] (Table 1).

Recently, a strategy to rapidly narrow down herbicidal chemicals from *Eucalyptus* EOs has been developed [74]. The strategy was validated for fast determination of the chemicals that take part in powerful herbicidal activities, based on gas chromatography–mass spectrometry (GC–MS) coupled with principal component analysis (PCA) and corroborated by the results of bioassays using different individual compounds [74]. In this work, the herbicidal activity of 17 EOs from 14 *Eucalyptus* species and hybrids was tested against *Lolium rigidum* Gaud. (Table 1), and two strong herbicidal compounds were determined using the developed strategy, trans-pinocarveol and α-terpineol. The herbicidal activity of trans-pinocarveol was revealed for the first time. The main compounds that constituted the *Eucalyptus* EOs tested (except for *E. grandis*) were 1,8 cineole and α-pinene, which have been reported to have bioactivities [80,81,82,83]. However, these two compounds did not explain the differences observed in the inhibitory activity of *L. rigidum* germination between the EOs. The authors concluded that other compounds, including minor components, could take part in the inhibitory activity of these *Eucalyptus* EOs. Among the main compounds determined as responsible for contributing to the strong herbicidal activity there were the terpenoids: borneol, pinocarvone, camphene, *exo*-fenchol, *trans*-*p*-mentha-1(7),8 dien-2-ol, α-terpineol, (Z)-ocimenone, epiglobulol, 2,2,5,5-tetramethyl-4-(2-hydroxy-2-methylbutylidene) cyclopenta-1,3-dione, myrtenol, *trans*-pinocarveol, (E)-caryophyllene, α-campholenal, *trans*-carveol, 6-camphenone and leptospermone.

Other works reporting EOs constituents, biological activity and main results have been included in Table 1.

One of the main difficulties that must be faced when testing the herbicidal activity of EOs is that they cannot be mixed with water, so emulsifiers are needed. In the majority of works, when EOs are tested in in vitro assays, they are applied directly to the paper in the Petri dishes. In other studies, different emulsifiers have been used, as Tween 20 [58,77], Tween 80 [84], acetone [60,61] or Fitoil [69,71,72]. When testing EOs in in vivo assays, it is necessary to prepare a solution of the EOs to apply them, so an emulsifier or an applicable formulation of the EOs is always needed. Another handicap, when testing herbicidal activity of EOs in in vivo conditions, is the rapid volatilization of EOs. This could be solved with a proper formulation of EOs. The commercially used formulations that are employed for EOs are microencapsulation and nanoemulsion [85].

Encapsulation is a method through which scientists mimic nature (is like imitating the membranes of cells or mitochondria). With the encapsulation the active ingredients are isolated, protected and can be functionally released [86]. Nanoencapsulation contributes to improving the bioavailability of the payload compounds, while enabling their controlled release and target delivery [85]. Hazrati et al. [67], as aforementioned, tested the herbicidal activity of a nanoemulsion of *S. hortensis* EO, which was prepared via low energy method using 96% (*v*/*v*) water, 2% (*v*/*v*) EO and 2% (*v*/*v*) Tween 80.

## 4. Effects of EOs on Plant Physiology and Mode of Action of Their Isolated Constituents

EOs are a complex mixture of biological active compounds, which can synergistically, additively and/or antagonistically interact among them [12]. Moreover, it should be considered that their composition is extremely variable depending on genotype, ecotype, biotic and abiotic stress, environmental condition and/or cropping management [90,91]. The complexity of the mixture joined to its high variability makes the identification of their mode of action extremely complex and hardly achievable.

On the contrary, despite the complexity and the necessity of a multidisciplinary approach, the identification of the mode of action of their pure constituents in controlled conditions is a simpler task. This is the main reason why we find many works in the literature focused on the mode of action of individual compounds rather than essential oils. Regardless, it should be highlighted that despite the lack of information concerning the EOs mode of action, several commercial organic herbicides are already available on the market (Section 5), whereas herbicides based on their pure constituents, which on the contrary have been deeply studied, are barely available.

### 4.1. Effects of EOs on Plant Physiology and Metabolism

To our knowledge, the only manuscript that has tried to hypothesize the mode of action of an EO was published by Araniti et al. [92]. Through an integrated physiological and metabolomic approach they were able to demonstrate that *Origanum vulgare* EOs treatment was able to inhibit the glutamate and aspartate metabolism altering the photorespiratory pathway and the photosynthetic machinery.

On the other hand, the literature reports much research focused on the potential application of EOs as leaf burning herbicides (with selective and/or nonselective activity) and on their effects on plant physiology.

Concerning the selectivity of the EOs, Synowiec et al. [93] reported that caraway EO emulsion was a good candidate for weed management in corn crops. This EO was selective, severely affecting the physiology and metabolism of *E. crus-galli* without damaging the corn. Moreover, the authors demonstrated that the application of this EO severely impacted the plant metabolism by altering several amino acidic pathways and the TCA cycle of treated plants. On the contrary, the same authors demonstrated that peppermint EO phytotoxicity was significantly higher than that of caraway EO but with no selectivity.

Concerning the effects of EOs on plant physiology, it was demonstrated that the application of different EOs, as *E. citriodora* [84], *E. tereticornis* [79] and *S. hortensis* [67], caused a reduction in the chlorophyll content and the cellular respiration of the treated weeds. These observations indicate that EOs were affecting negatively the photosynthetic system and the energy metabolism of the target plants [84].

Phytotoxic effects of EOs that can be visible in treated plants, like growth reduction, chlorosis and burning of leaves, have been attributed to their interference with some processes in the plant cells, like inhibition of mitosis, decrease of cellular respiration and chlorophyll content, membrane depolarization and ion leakage, removal of the cuticular waxes, oxidative damages and microtubule polymerization [24]. EOs that alter the membrane integrity (e.g., *E. citriodora* EO), consequently increasing its permeability and enhancing the solute leakage [75], affect other physiological and biochemical processes linked to membrane functioning [64].

Regarding the inhibitory effects on seed germination of EOs, it was demonstrated that the phytochemicals prevent seed germination by the disruption of mitochondrial respiration and oxidative pentose phosphate pathway (OPPP) [94,95,96].

The reduction that EOs cause on seedling root and shoot length can be attributed to the reduced rate of cell division and cell elongation due to the activity of allelochemicals and reduced mitotic index [97].

The main components of EOs are monoterpenes, but oxygenated monoterpenes have shown more phytotoxic effects than monoterpene hydrocarbons [57,98]. EOs and their components can cause anatomical and physiological changes in plant seedlings, causing accumulation of lipid globules in the cell cytoplasm and reduction in membrane permeability and respiration, possibly due to the inhibition of DNA and RNA syntheses [48]. Phytotoxicity can be increased with the synergistic effects of EO components [99].

### 4.2. Terpenoids Phytotoxicity and Mode of Action

The phytotoxic potential of terpenoids has been largely documented in the last 20 years. Because of their relatively simple structure, as well as their multiple biological activity and ecofriendly characteristics, they have been proposed for the formulation of natural herbicides or as a backbone for the production of synthetic natural-like herbicides [100]. Nevertheless, despite the extensive proof concerning their in vitro and in vivo (both microcosms and open field) phytotoxicity, only few experiments have highlighted, or at least have tried to highlight, their mode of action.

In the present review, we tried to focus on those molecules that have been deeply studied, giving a proof and/or a hint concerning their potential mode of action.

Amongst terpenoids, cineoles have been the most widely studied, and 1,4 cineole is of particular interest because of its similarity in structure with the synthetic herbicide cinmethylin [101]. This molecule, as well as several other terpenoids, significantly altered the mitotic process affecting the prophase. Moreover, it induced growth abnormalities in shoots, such as helical growth [81] (Table 2).

Similarly, 1,8-cineole (eucalyptol), a monoterpene largely produced by the allelopathic species *Eucalyptus* sp. [102], is a ubiquitous terpene characterized by a strong inhibition of plant growth affecting mitochondrial respiration and, in onion roots, inducing the formation of swollen root tips and the inhibition of all the mitosis stages [81,103] (Table 2).

Moreover, a recent publication [104] demonstrated that 1,8-cineole vapors were able to inhibit the tuber sprout growth of *Solanum tuberosum* ”Russet Burbank” reducing gibberellin production (in particular GA20 but not GA19). The authors, by supplying exogenous gibberellins (GA1, GA3 and GA20), were able to reverse cineole-induced sprout growth inhibition. In addition, the expression of genes encoding key gibberellin metabolic enzymes was significantly altered by the treatment, suggesting that this natural monoterpenoid interferes with plant growth and development by impairing the biosynthesis of gibberellin (Table 2).

The radial root tip swelling observed after 1,8-cineole treatment and the helical growth of the shoot of plants treated with 1,4-cineole are a clear sign that these molecules are able to interfere with cell division and with the cortical microtubule organization (Table 2). In fact, Baskin et al. [105] demonstrated that the chemicals oryzalin and colchicine, two tubulin disorganizers, could induce two different effects on the root tip: radial expansion (phenomenon known as swelling) or root growth inhibition, which can be accompanied by a characteristic anisotropic growth “corkscrew shape” (phenomenon known as handedness) [106]. In particular, Baskin et al. [105] demonstrated that the root swelling is mainly due to a disorganization of cortical microtubules, which stimulated the tangential expansion and reduced the uniformity of cellulose microfibril alignment among cells.

Although no evidence has been provided regarding the effects of 1,8-cineole on microtubules, the ability of terpenoids, in particular mono- and sesquiterpenes, in altering both cortical and spindle and phragmoplast microtubules, has been largely documented in the last few years.

The first evidence, concerning microtubules as intracellular target of the terpenoids, was given by Chaimovitsh et al. [107] with the monoterpene citral. In a first experiment, they were able to demonstrate that a few minutes exposition of *Arabidopsis* seedlings to citral vapors was sufficient to disrupt the microtubules organization and polymerization, leaving intact the actin filaments (Table 2).

Successively, working on wheat roots, they further demonstrated that citral treatment led to the alteration of root growth and cell ultrastructure (curvature of newly formed cell walls and deformation of microtubule arrays) [108]. They highlighted that mitotic microtubules were more sensitive than cortical and, as a consequence, citral was able to disrupt mitotic microtubules, inhibiting the cell cycle and increasing the frequency of asymmetric cell plates in treated cells by directly interfering on cell plates during their formation [107,108] (Table 2).

More recently, Graña et al. [109] were able to further confirm the citral-mediated inhibition of cell division and to link for the first time the ultrastructure cellular alteration to a biased hormonal balance (auxin and ethylene), and to a reduction in plasmodesmata-mediated cell–cell communication (Table 2). In addition, through a staining technique the authors have been able to histolocalize citral accumulation in *Arabidopsis* roots demonstrated that it accumulates mainly in the differentiation zone [110] (Table 2).

Finally, in 2020, through a transcriptomic and molecular docking approach, Graña et al. [111] demonstrated that just 1 h of exposition to citral was enough to induce an inhibition of the single strand DNA-binding proteins, as a consequence, downregulating the genes transcription (Table 2).

A link between cell division, microtubule organization and hormonal level was also highlighted by Araniti et al. [112,113] with the sesquiterpene farnesene. This ubiquitous molecule, generally present in low concentrations in various plant species, has been shown to play a fundamental role in the defense of the plant, and the increase in its production is closely linked to the presence of stress [114,115]. Araniti et al. [113] demonstrated that farnesene was inducing reactive oxygen species (ROS) accumulation, alteration of cell division (bi- and tetranucleated cells) and an anisotropic growth of the root (left handedness and loss of the gravitropic response), mainly due to auxin-mediated microtubule malformations (Table 2). Further investigating this phenomenon, they were able to demonstrate that the microtubule malformation was a consequence of an altered auxin distribution. In fact, during their experiments they observed that farnesene was able to downregulate all the auxin polar transport proteins (PIN proteins) and, through a confocal microscopy approach using GFP (green fluorescent protein) mutant lines, they were able to observe a complete absence of the proteins PIN4 and PIN7, pivotal for auxin redistribution in root meristem, at the level of the quiescent center [112] (Table 2). Furthermore, they demonstrated anatomically that the left-handedness phenotype was due to a difference in length between the inner cell of the root meristem and the epidermal cells. In particular, because of farnesene-mediated microtubule malformations, the inner cells were shorter than epidermal cells and the last were characterized by an abnormal shape [112].

In addition, the sesquiterpene alcohol nerolidol (Table 2) induced in *A. thaliana* an alteration in root morphology, growth and development, altering the auxin balance and inducing ROS accumulation followed by lipid peroxidation. Moreover, as observed in farnesene, the primary root was characterized by a random corkscrew shape indicating a possible alteration of the cortical microtubules. On the other hand, plants were able to counteract the stress by increasing the activities of the two ROS scavengers, superoxide dismutase and catalase, as well as inducing the production of metabolites with osmoprotectant activity. The metabolomic pathway analysis highlighted that starch and sucrose metabolism, alanine, aspartate and glutamate metabolism, and glycine, serine and threonine metabolism were the most affected pathways [116] (Table 2).

Similar to farnesene, Yan et al. [117] reported that artemisinin, a sesquiterpene endoperoxide, was inducing in lettuce root tips an arrestment of cell division, and the loss of cell viability because of an artemisin-induced ROS burst followed by an increase of lipid peroxidation and damages to cell membrane. Successively, they also demonstrated that the same molecule altered the root gravitropic responses, as well as root growth, development and architecture, in seedlings of *Arabidopsis thaliana* They further demonstrated that the gravitropic alterations observed were mainly due to a reduction in the number of starch grains in the columella cells and to altered auxin lateral distribution (inhibition of the PIN2 carrier) [118] (Table 2).

All those findings are extremely interesting since the ability of terpenoids to inhibit and/or alter mitosis and cell division is a phenomenon that has been largely reported in the past [81,108,109,119], but only now it is possible to affirm that such alterations are the consequence of the direct effects of these molecules on microtubule organization, suggesting that terpenoids, at least those which are able to affect mitosis, can be considered as a class of mitotic disrupter bioherbicides [120].

However, in vitro experiments are pivotal for a rapid and economic screening of molecule phytotoxicity, for the identification of the main target (root and shoot), for the identification of key concentrations (e.g., effective and lethal doses), and for the potential identification of their mode of action under controlled conditions; often the effects observed in vitro on seedlings do not coincide with those observed on adult plants of the same species [109,121,122]. Therefore, it is usually necessary to increase the concentrations of the molecules assayed to observe phytotoxicity effects.

For example, the terpenoid citral was extremely active in vitro at concentrations of 194 µM (IC_50_—dose necessary to inhibit the root length by 50%), whereas it was necessary to increase its concentration at least 10 times to see phytotoxic effects on adult plants [121,122].

In particular, they observed that the application of citral through irrigation or spraying had completely different effects on plants, suggesting that foliar or root absorption can determine the effectiveness of this compound. Anyway, the subirrigation method was the most effective, inducing an alteration of the plant water status followed by oxidative stress and damages to the photosynthetic machinery, which resulted in a strong reduction of plant growth and development [121] (Table 2). The same authors, further investigating the effects of this molecule on the plant water balance, observed a time dependent reduction of stomatal conductance accompanied by a reduction of leaf relative water content and its water potential. Moreover, the plant fitness was extremely compromised since treated plants were unable to complete their leaf cycle because of a premature withering of the flowers and the inability to produce silique and develop seeds [122] (Table 2). These effects are extremely encouraging since citral application, if its effects are reproducible on weed species, could be used as an ecofriendly herbicide able to reduce the fitness of the crop competitors, without completely eradicating them (allowing the maintenance of the biodiversity in the agroecosystem), and to strongly reduce the weed seed bank in the soil.

In addition to mitosis, the alteration of the plant water status is a phenomenon commonly observed in adult plants treated with terpenoids. For example, it was reported that the lipophilic layers of the leaf surface and the stomata are primary targets of menthol and camphor. Full expanded rosettes of *Arabidopsis* exposed for different hours to monoterpenes vapors were characterized by an increase of stomatal aperture, followed by an extreme swelling and a final breakdown of the protoplasts, an alteration of cuticular wax layer, which induced, as a consequence, huge water loss and plant death [123]. They further demonstrated that both compounds, in particular camphor, prevented stomatal closure and inducing changes to stomata cytoskeleton, which plays a pivotal role in stomatal movements. Moreover, the prolonged treatment induced a reduction of the expression of the genes MPK3 and ABF4, which encode for proteins involved in the process of stomatal closure, and a concomitant downregulation of the PEPCase expression, which is an enzyme important for stomatal opening. All these effects were followed by an alteration of the plant water status accompanied by a reduction in growth and development, and a final plant death if the exposure to vapors was prolonged for more than 48 h [124,125] (Table 2).

Araniti et al. [125] observed that adult plants of *Arabidopsis* treated through irrigation with the sesquiterpene *trans*-caryophyllene were characterized by pinwheel shaped rosettes (Table 2). In addition, this phenomenon is known as handedness since it could interest both root and shoot organs. Regardless, as for the roots, it is due to a microtubular alteration and a disturbance to their dynamic instable equilibrium. Such a phenomenon was observed in plants subjected to salinity stress [126]. As for salinity stressed plants, and plants treated with this sesquiterpenoid were characterized by a significant alteration of the plant water status accompanied by an accumulation of reactive oxygen species and lipid peroxidation, which led to physical damages in the PSII antenna complexes and a consecutive reduction of carbon assimilation [125].

Similarly, in a recent experiment, the terpenic phenol thymol caused a significant alteration of the plant water status accompanied by increase in ABA (abscisic acid) content, which induced a total closure of the stomata and the accumulation of heat at the level of the leaf lamina. These effects were followed by a strong accumulation of reactive oxygen species and damages to the photosynthetic machinery. The authors demonstrated that the plants were activating several biochemical and metabolic mechanisms aimed to counteract the stress. In particular, the thymol-treated adult plants increased the production of several osmoprotectants metabolites (quaternary ammonium compounds, hexoses, polyols, anthocyanins etc.) and upregulated the production of several proteins involved in ROS scavenging activities. On the other hand, several proteins involved in photosynthesis were significantly downregulated by the treatment (e.g., the light harvesting complexes of PSII (LHCIIs), the PSII reaction centers (RCIIs), antenna pigment-protein complex CP47 etc.) [127] (Table 2).

Other molecules, belonging to the terpenoids class, were characterized by a mode of action, which reduced pigment content and/or impaired the PSII system. Among them, the sesquiterpene endoperoxide artemisinin should be mentioned. Bharati et al. [128] demonstrated that this molecule was able to inhibit the electron transport activity in chloroplasts affecting, as target site, the secondary quinone moiety of photosystem II complex (QB). Moreover, they suggested that the phytotoxicity of this molecule at PSII level was mainly due to the formation of a complex between the molecule and an protein known as “herbicidal binding protein,” which is known to bind to several herbicide and natural compounds such as atrazine, fisherellin, grandinol, sorgoleone etc., impairing the electron flow beyond the primary electron-accepting plastoquinone QA (Table 2).

More recently, Hussain and Reigosa [129] further confirmed that artemisinin, in adult plants of *Arabidopsis*, altered the photosynthetic machinery, reducing the photosynthetic efficiency, PSII yield and the electron transport rate (Table 2).

## 5. Commercial Herbicides Based on EOs

Among the commercial organic herbicides based on EOs and/or their compounds, mainly available in the USA market, these should be mentioned: herbicides based on clove EO (*S. aromaticum*); summer savory EO (*S. hortensis*); cinnamon EO (*C. zeylanicum*); red thyme EO (*Thymus zygis* L.); lemongrass EO (*Cymbopogon citratus* (DC.) Stapf); d-limonene, one of the major compounds in many citrus EOs, as orange (*Citrus sinensis* (L.) Osbeck), lemon (*C. limon*) or mandarin (*Citrus reticulata* Blanco); and eugenol, the main compound of clove EO. The commercial products available are GreenMatch (55% d-limonene), Matratec (50% clove oil), WeedZap (45% clove oil + 45% cinnamon oil), GreenMatch EX (50% lemongrass oil), Avenger Weed Killer (70% d-limonene) and Weed Slayer (6% eugenol) [130,131]

## 6. Conclusions and Future Perspectives

In recent years, research regarding the herbicidal potential of EOs and their constituents has strongly increased. The companies involved in marketing phytosanitary products are becoming more interested in natural products for integrated pest management, and they are investing in the research of natural products as bioherbicides. EOs, because of their high phytotoxic activity, are promising candidates for the development of new potentially ecofriendly products. The main challenges are (i) to develop adequate formulations that would allow applying phytotoxic EOs under field conditions, minimizing their high volatility and enhancing their penetrability in plants; (ii) the difficulty and high cost of registering them in the European market as phytosanitary products, due to their mix of compounds; and (iii) to determine their mode of action, since EOs are a complex mixture of biologically active compounds able to affect several targets at the same time, which may be valuable for avoiding the development of resistant weeds. This could help also to better understand how they work and, consequently, improve their formulation and application.

Identifying the mode of action of pure isolated terpenoids is growing faster since the main experiments are carried out under controlled conditions using a single experimental factor (pure compound concentration), which gives the possibility of attributing to a single molecule the morphological, physiological, metabolic and molecular variations induced in the treated species. Moreover, the use of new *-omics* approaches (genomic, transcriptomic, proteomic and metabolomic) joined to the classical analytical techniques is giving new cues and hints, which is speeding up the workflow for the study and discovery of new mechanisms of action.

In the future, the knowledge about terpenoids with known mechanisms of action would allow the development of natural herbicides, which could exploit the potential synergism among single molecules, thus reducing the application doses, and still having herbicidal products capable of simultaneously hitting multiple targets. Moreover, this will help to avoid the development of resistance.

All the advances achieved suggest that in the next years new bioherbicides based on EOs or on their constituents will be also produced in the European market.

## Figures and Tables

**Table 1:** Reported herbicidal activity of essential oils.

EO Tested	Main Compounds of the EO	Species against Which the EO Have Been Tested	Methodology	Herbicidal Activity/Phytotoxic Effects Observed	Bibliography
32 EOs preliminary tested *Micromeria fruticosa* *Artemisia judaica* (Israeli var.) *Mentha piperita* *Cymbopogon citratus* *Artemisia judaica* (Sinai var.) *Mentha longifolia* *Melissa officinalis* *Salvia officinalis* (Dalmatian var.) *Eucalyptus citriodora* *Ocimum citriodorum* *Rosmarinus officinalis* *Artemisia arborescens* *Carun carvi* *Lavandula officinalis* *Thymus citriodorus* *Ocimum basilicum* *Hyssopus officinalis* *Coriandrum sativum* *Coridothymus capitatus* *Origanum syriacum* (thymol chemotype) *Origanum vulgare* (thymol chemotype) *Origanum majorana* *Lippia citriodora* *Origanum syriacum* (carvacrol chemotype) *Thymus vulgaris* *Myrtus communis* *Laurus nobilis* *Pelargonium graveolens* *Ocimum basilicum* (methyl chavicol type) *Artemisia dracunculus* (Russian var.) *Artemisia dracunculus* (French var.) *Artemisia absinthium*	Not reported	*Triticum aestivum*	*In vitro* germination inhibition test in Petri dishes with filter paper.	50% inhibition of *T. aestivum* germination for 28 EOs (doses 28–84 nL/mL), 4 EOs were not active: *O. basilicum* (Methyl chavicol type), *A. dracunculus* (Russian var.), *A. dracunculus* (French var.), *A. absinthium*; 3 EOs selected for further studies: *O. syriacum*, *C. citratus* and *M. fruticosa*.	[57]
*Origanum syriacum* *Cymbopogon citratus* *Micromeria fruticosa*	Carvacrol (60.1%) Citral (geranial 42.6% and neral 32.1%) Pulegone (59.7%)	*Triticum aestivum*	*In vitro* germination inhibition test in Petri dishes with filter paper.	*C. citratus* EO was the most effective: 50% inhibition of *T. aestivum* germination at 32 nL/mL and 50% inhibition of *T. aestivum* radicle growth at 16 nL/mL.	[57]
*Origanum syriacum* *Cymbopogon citratus* *Micromeria fruticosa*	Carvacrol (60.1%) Citral (geranial 42.6% and neral 32.1%) Pulegone (59.7%)	*Amaranthus blitoides* *Amaranthus palmeri* *Euphorbia hirta* *Sinapis nigra* *Trifolium campestre* *Solanum lycopersicum*	*In vitro* germination inhibition test in Petri dishes with filter paper.	Different sensitivity depending on the species (IC_50_ doses from 8 to 116 nL/mL).	[57]
*Origanum syriacum* *Cymbopogon citratus* *Micromeria fruticosa*	Carvacrol (60.1%) Citral (geranial 42.6% and neral 32.1%) Pulegone (59.7%)	*Triticum aestivum* *Amaranthus palmeri*	Germination and growth inhibition test in clayey soil, doses 0.5–2%.	The most active EO was *C. citratus* (at 2%, 100% inhibition of *T. aestivum* germination, 92% inhibition *A. palmeri* germination). Different sensitivity to the EOs depending on the species.	[57]
*Cymbopogon citratus*	Citral (geranial 42.6% and neral 32.1%)	*Triticum aestivum* *Brassica nigra* *Amaranthus palmeri*	Germination and growth inhibition test in clay soil, loam and loess, doses 0.5–2%.	Different sensitivity depending on the species. The EO was more effective inhibiting germination in loam or loess than in clayey soil. Germination inhibition decreased with increase of sowing depth.	[57]
*Ocimum basilicum* *Salvia sclarea* *Carum carvi* *Melissa officinalis* *Coriandrum sativum*	Not reported	*Ocimum basilicum* *Salvia sclarea* *Carum carvi* *Melissa officinalis* *Triticum aestivum* *Sinapis nigra* *Solanum lycopersicum*	*In vitro* germination inhibition test in Petri dishes with filter paper.	Different sensitivity depending on the species and the EO tested (IC_50_ doses (dose necessary to inhibit the germination by 50%) from 12 to 108 nL/mL). *O. basilicum*, *S. sclarea*, *C. carvi* and *M. officinalis* EOs inhibited the germination of seeds of the plant from which they were extracted.	[57]
*Ocimum basilicum* *Brassica napus* *Cinnamomum zeylanicum* *Carum carvi* *Syzygium aromaticum* *Zea mays* *Gossypium hirsutum* *Vaccinium macrocarpon* *Foeniculum vulgare* *Linum usitatissimum* *Vitis amurensis* *Corylus avellana* *Simmondsia chinensis* *Limnanthes alba* *Olea europaea* *Arachis hypogaea* *Prunus* spp. *Brassica napus* *Carthamus tinctorius* *Sesamum indicum* *Glycine max* *Satureja hortensis* *Helianthus annuus* *Betula nigra* *Thymus vulgaris*	Not reported	*Taraxacum officinale*	Phytotoxic effects of the EOs were evaluated in separated dandelion leaves in laboratory experiments (doses tested 0, 0.5, 1.0 and 2.0%, *v*/*v*, EOs prepared in mineral oil).	The most effective EOs were *Thymus vulgaris*, *Satureja hortensis*, *Cinnamomum zeylanicum* and *Syzygium aromaticum* (doses 1% *v*/*v*). They all caused electrolyte leakage resulting in cell death.	[20]
*Thymus vulgaris* *Satureja hortensis* *Cinnamomum zeylanicum* *Syzygium aromaticum*	Not reported Not reported Not reported Eugenol (84% *v*/*v*)	*Chenopodium album* *Ambrosia artemisiifolia* *Sorghum halepense*	*In vivo* assays to test EOs against weeds (whole plants) in greenhouse conditions. EOs were prepared in aqueous solutions (5–10% *v*/*v*) with two adjuvants, a nonionic surfactant and paraffinic oil blend at 0.2% *v*/*v*, and were applied on shoots.	Shoot death was verified within 1 h and 1 day after treatment. *S. aromaticum* was the most active EO.	[20]
*Rosmarinus officinalis* ecotype A *Rosmarinus officinalis* ecotype B *Thymus vulgaris* *Satureja montana*	α-Pinene (37.2%) and 1,8-cineole (22.6%) α-Pinene (13.5%), 1,8-cineole (46.8%), borneol (12.9%) Thymol (44.1%) Carvacrol (56.8%)	*Chenopodium album* *Portulaca oleracea* *Echinochloa crus-galli* *Raphanus sativus* *Capsicum annuum* *Lactuca sativa*	Germination and growth inhibition tests in Petri dishes with filter paper, EOs prepared in an aqueous solution (500 mg/L) with Tween 20 (100 mg/L).	*S. montana* was the most effective EO, inhibiting germination of all species tested. *T. vulgaris* activity was more selective, depending on the species on which it was applied. *R. officinalis* A caused germination inhibition and abnormal seedlings. *R. officinalis* B showed greater herbicidal activity than A.	[58]
			Pure compounds carvacrol, thymol, borneol and 1,8-cineole were also tested, prepared in aqueous solutions (250 mg/L) with Tween 20 (100 mg/L).	Carvacrol was the most active pure compound, completely inhibiting germination in all species except radish.	
*Eucalyptus citriodora*	Not reported	*Triticum aestivum* *Zea mays* *Raphanus sativus* *Cassia occidentalis* *Amaranthus viridis* *Echinochloa crus-galli*	*In vitro* germination and growth inhibition tests in Petri dishes were carried out separately. Doses tested: 0.03, 0.06, 0.12, 0.30, 0.60 and 1.20 mg/L for germination inhibition tests and 0.12 and 0.30 mg/L for growth inhibition tests.	The germination of all species was reduced significantly at concentrations ≥0.30 mg/L, species demonstrated different sensibility to the EO. *A. viridis* was the most sensitive species while *Z. mays* and *R. sativus* were the most resistant. Seedling growth was also affected by EO application. The total chlorophyll content and the respiratory activity of treated seedlings were strongly reduced. *A. viridis* was again the most sensitive species.	[84]
*Eucalyptus citriodora*	Not reported	*Echinochloa crus-galli* *Cassia occidentalis*	*In vivo* experiments in greenhouse, post-emergence assays in 4-weeks-old weeds. Doses tested: 2.5%, 5.0% and 7.5% solution of EO in water, applied by spraying.	Chlorophyll content and respiratory activity were affected in both weeds. *C. occidentalis* respiratory activity and chlorophyll content were reduced by 85% when sprayed with 2.5% solution of EO.	[84]
*Eucalyptus citriodora*	Not reported	*Echinochloa crus-galli* *Cassia occidentalis*	Field experiment in parcels of 1 × 10 m, where weed species were sown. Doses applied by spraying: 1%, 2.5%, 5.0% and 10% of EO, prepared in water with the help of surfactant Tween- 80 at concentration 0.05% *v*/*v*.	At low concentrations (0.5% and 1%), few effects were observed. At 7.5% and 10%, *C. occidentalis* was completely eliminated 1 day after treatment. The injury level on *E. crus-galli* at these doses was 50–76%.	[84]
*Eucalyptus citriodora*	Not reported	*Parthenium hysterophorus*	Laboratory bioassay in Petri dishes, germination and growth inhibition test. Doses assayed: 0.20, 0.50, 1.0, 2.0 and 5.0 nL/mL.	Seed germination, seedling length, chlorophyll content and respiratory activity of the weed were reduced with increasing EO concentration. Germination was completely inhibited at 5.0 nL/mL.	[75]
			*In vivo* assay in 4-week-old plants. Doses tested: 0, 5, 25, 50, 75 or 100 µL/mL.	Damage symptoms increased, while chlorophyll content and the respiratory activity decreased with increased EO concentrations. Up to 50 µL/mL some plants recovered over time, but plants sprayed with 75 and 100 µL/mL died 2 weeks after treatment application. Treated plants suffered a rapid electrolyte leakage at concentrations between 5–75 µL/mL, indicating an effect on membrane integrity.	
*Hyssopus officinalis* *Lavandula angustifolia* *Majorana hortensis* *Melissa officinalis* *Ocimum basilicum* *Origanum vulgare* *Salvia officinalis* *Thymus vulgaris*	Not reported	*Raphanus sativus* *Lactuca sativa* *Lepidium sativum*	Germination and growth inhibition assays in Petri dishes. EOs were prepared in water–acetone mixture (97.5:2.5), and were tested at doses: 2.5, 1.25, 0.625, 0.25, 0.125 and 0.06 μg/mL.	At the lowest dose some of the tested EOs were able to promote both germination and radical elongation. At the highest dose, all EOs except *O. basilicum* inhibited completely the germination of *R. sativus*. *L. sativa* germination was completely inhibited by *M. officinalis*, *M. hortensis*, *O. vulgare* and *T. vulgaris* EOs. *L. sativum* was 100% inhibited by *H. officinalis*, *M. officinalis*, *O. vulgare* and *T. vulgaris*.	[60]
			Effects on germination of EO vaporization: 1.5 mL of EO in each Petri dish.	All the EOs showed strong inhibitory activity on both germination and growth of the 3 species tested, with inhibition values above 72%	
*Cinnamomum zeylanicum* *Lavandula* spp. *Mentha* × *piperita*	Not reported	*Amarantus retroflexus* *Sinapis arvensis* *Lolium* spp.	*In vivo* assays in pots, in greenhouse. Oil-in-water emulsion was prepared at the doses: 5.4, 21.6, 86.4 and 345.6 mg/L; 5 mL were sprayed on the soil surface of each pot after sowing. Control pots were irrigated with water.	Application of EOs reduced weed germination at all concentrations. *C. zeylanicum* was the most active EO. The highest concentration tested of cinnamon and lavender EOs controlled significantly germination of all weeds. *Lolium* spp. germination was reduced by 52% with *C. zeylanicum* EO and by 51% with *Lavandula* spp. EO. *S. arvensis* germination was inhibited 79% by *C. zeylanicum* EO and 58% by lavender EO, while *A. retroflexus* did not germinated with the maximum dose of *C. zeylanicum* EO and its germination was reduced by 85% with lavender EO. *M. piperita* EO showed the maximum inhibitory effect for *A. retroflexus* at the highest dose applied (82% of germination inhibition) and for the other species at the third dose tested (62% germination reduction for *Lolium* spp. and 44% for *S. arvensis*). The dicotyledonous species were more susceptible to the EOs compared with the monocotyledonous.	[68]
*Eucalyptus citriodora*	Not reported	*Bidens pilosa* *Amaranthus viridis* *Rumex nepalensis* *Leucaena leucocephala*	*In vitro* germination and growth inhibition assays in Petri dishes. Doses tested: 0.0012 to 0.06%.	The EO reduced the germination and seedling growth of the weeds. At 0.06% no weed seed germinated. *A. viridis* was the most sensitive species, with its germination completely blocked at 0.03% dose. The chlorophyll content and the respiratory activity of the leaves of emerged seedlings were also affected. *A. viridis* chlorophyll content and respiratory activity were reduced by 51 and 71%, respectively, at 0.06% dose.	[76]
*Eucalyptus camaldulensis* *Lantana camara* *Eriocephalus africanus*	Spathulenol (41.46 ± 3.04%), p-cymene (21.92 ± 1.61%), cryptone (7.76 ± 0.62%) α-Curcumene (23.09 ± 2.10%), γ-curcumene (14.64 ± 1.06%), γ-muurolene (12.54 ± 1.43%) Artemisia ketone (56.46 ± 1.99%), intermedeol (9.59 ± 0.89%)	*Amaranthus hybridus* *Portulaca oleracea*	*In vitro* assays in Petri dishes, germination and seedling growth inhibition test. Doses assayed: 0.125, 0.25, 0.5 and 1 µL/mL.	*E. camaldulensis* EO was the most effective, completely controlling the germination and seedling growth of both weeds. *E. africanus* EO was very effective against *A. hybridus* germination but only reduced slightly *P. oleracea* germination at the two highest doses tested. *L. camara* inhibited *A. hybridus* germination and seedling length, but showed no effect against *P. oleracea* germination, although reduced its seedling growth.	[73]
*Cinnamomum zeylanicum* *Lavandula* spp. *Mentha* × *piperita*	Not reported	*Amaranthus retroflexus* *Solanum nigrum* *Portulaca oleracea* *Chenopodium album* *Sinapis arvensis* *Lolium* spp. *Vicia sativa*	For each EO, an oil-in water emulsion was prepared. In vitro assays, germination inhibition tests in Petri dishes. EO concentrations tested: 0.2, 0.6, 1.8 and 5.4 mg/L. Water was used in controls.	EOs inhibited weed seed germination. The concentration to reach 100% germination inhibition was different for each EO and depended on the species tested. *C. zeylanicum* was the most active EO. *A. retroflexus*, *P. oleracea* and *V. sativa* were the most sensitive weeds, as all EOs inhibited their germination at 1.8 mg/L. *S. arvensis* and *Lolium* spp. were the more resistant weeds, only being completely inhibited at the highest concentration (5.4 mg/L).	[63]
			*In vivo* assays in pots, in greenhouse. EO concentrations tested: 5.4, 21.6, 86.4 and 345.6 mg/L.	*C. zeylanicum* EO was the most active. At the maximum concentration inhibited completely the germination of *A. hybridus*, reducing the germination of *S. arvensis* by 78% and decreasing *Lolium* spp. germination by 57%.	
*Hyssopus officinalis* *Lavandula angustifolia* *Majorana hortensis* *Melissa officinalis* *Ocimum basilicum* *Origanum vulgare* *Salvia officinalis* *Thymus vulgaris* *Verbena officinalis* *Pimpinella anisum* *Foeniculum vulgare* *Carum carvi*	*iso*-Pinocamphone (29.1%), β-pinene (18.2%), *trans*-pinocamphone (11.2%) Linalool (23.1%), linalyl acetate (44.4%), geraniol (9.3%) 1,8-Cineole (33.5%), linalool (9.8%), α-pinene (9.0%) (-)-Citronellal (39.6%), carvacrol (13.3%), *iso*-menthone (8.8%) *iso*-Pinocamphone (35.1%), carvone (39.7%) Carvacrol (44%), o-cymene (41.9%) *trans*-Thujone (37.9%), camphor (13.9%), borneol (7.6%) *o*-Cymene (56.2%), carvacrol (24.4%), thymol (8.7%)Isobornyl formate (45.4%), (E)-citral *cis*-Anethole (97.1%) *cis*-Anethole (76.3%), fenchone (14.2%) Estragole (65.0%), limonene (14.3%)	*Raphanus sativus Lactuca sativa Lepidium sativum*	Germination and growth inhibition assays in Petri dishes. EOs were prepared in water–acetone mixture (99.5:0.5), assayed at doses: 2.5, 1.25, 0.625, 0.25, 0.125 and 0.06 μg/mL.	All EOs were active against germination and early radicle growth of the three species tested, showing different levels of activity, with the most active being *T. vulgaris*, *M. officinalis*, *V. officinalis* and *C. carvi* EOs. *L. sativum* was the less sensitive seed. All EOs tested, except *P. anisum*, *O. basilicum* and *F. vulgare*, inhibited by 100% the germination of *R. sativus*, at the highest dose tested, while *M. officinalis*, *C. carvi*, *H. officinalis*, *T. vulgaris* and *V. officinalis* inhibited 100% *L. sativum* germination at the highest dose. *T. vulgaris* and *O. vulgare* EOs inhibited both germination and radicle elongation of *L. sativum* at 1.25 μg/mL. *C. carvi*, *V. officinalis*, *S. officinalis* and *M. hortensis* EOs affected, significantly the radicle elongation of *L. sativum* at all doses. *P. anisum* was the less active EO on germination, whereas *F. vulgare* EO was less active on radicle elongation of *L. sativum*. Some EOs (*P. anisum* and *O. basilicum*) stimulated the germination and/or radicle elongation of *L. sativum* at the lowest dose. *V. officinalis* EO inhibited by 100% the germination of *R. sativus*, at almost all doses tested. *C. carvi*, *H. officinalis* and *S. hortensis* EOs inhibited significantly the germination of *R. sativus*, at all doses tested. The radicle growth of *R. sativus* was affected by 100% by *V. officinalis*, *C. carvi*, *O. vulgare*, *T. vulgaris, H. officinalis* and *L. angustifolia* EOs at the three highest doses assayed. All these EOs, except *L. angustifolia* EO, were active towards radicle elongation, at all doses. *T. vulgaris* EO inhibited by 100% germination and radicle elongation of *L. sativa* seeds, at all assayed doses. *V. officinalis*, *M. officinalis* and *C. carvi* EOs inhibited significantly germination of *L. sativa* seeds at all doses. *M. hortensis* and *V. officinalis* inhibited significantly the radicle growth of *L. sativa* seeds. *F. vulgare* and *P. anisum* were among the less active EOs against *L. sativa*.	[61]
*Artemisia scoparia*	*p*-Cymene (20.5%) β-myrcene (13.95%) (+)-limonene (12.53%)	*Achyranthes aspera* *Cassia occidentalis* *Parthenium hysterophorus* *Echinochloa crus-galli* *Ageratum conyzoides*	Bioassay carried out in Petri dishes filled with sand impregnated with *A scoparia* EO at 5, 10, 25 and 50 μg oil/g sand.	The germination and seedling growth (root and shoot length) were significantly reduced at doses ≥10, 25 and 50 μg *A. scoparia* EO/g sand. The effect was greater on root length than on shoot length. The most sensitive species was *P. hysterophorus* followed by *A. conyzoides* and the most resistant was *C. occidentalis.*	[64]
			*In vivo* experiment in a greenhouse on 6-week-old weed plants raised under controlled conditions, sprayed with 2, 4 and 6% (*v*/*v*) solution of *A. scoparia* EO on distilled water.	EO application provoked visible injury symptoms (1 and 7 days after spray) extending from chlorosis to necrosis to complete wilting of plants. The most sensitive weeds were *E. crus-galli* and *P. hysterophorus*. The treatment with EO caused loss of chlorophyll content and cellular respiration in tested weeds, suggesting an interference/impairment with photosynthetic and respiratory metabolism. The EO produced a severe electrolyte leakage on *E. crus-galli* (a monocot) and *C. occidentalis* (a dicot) indicating membrane disruption and loss of integrity.	
*Peumus boldus* *Drimis winteri*	Ascaridole (51.17 ± 9.51%), p-cymene (16.31 ± 2.52%) and 1,8-cineole (14.45 ± 2.99%) γ-Eudesmol (21.65 ± 0.41%), elemol (12.03 ± 0.34%), terpinen-4-ol (11.56 ± 1.06%)	*Amaranthus hybridus* *Portulaca oleracea*	*In vitro* experiments in Petri dishes, germination and growth inhibition test. Doses tested: 0.125, 0.25, 0.5, 1 μL/mL.	*P. boldus* was the most active EO, inhibiting seed germination and growth of both species tested. *D. winteri* only affected *P. oleracea* germination at the highest dose applied.	[21]
*Cistus ladanifer*	*trans*-Pinocarveol (20.00%), viridiflorol (13.59%), bornyl acetate (7.03%)	*Amaranthus hybridus* *Portulaca oleracea* *Chenopodium album* *Conyza canadensis* *Parietaria judaica*	*In vitro* germination and growth inhibition assays in Petri dishes. Doses tested: 0.125, 0.25, 0.5, 1 μL/mL.	*A. hybridus* was the most sensitive species; its germination was completely blocked at all concentrations. *C. canadensis* and *P. judaica* were also very sensitive, their germination was almost completely controlled. *P. oleracea* germination was inhibited at the two higher doses tested, and *C. album* was the most resistant weed, no effect was observed on its germination. The EO showed a strong phytotoxic activity on seedling length of all weeds at all concentrations tested.	[66]
*Melaleuca armillaris* *Melaleuca styphelioides* *Melaleuca acuminata*	*cis*-Calamenene (19%), torreyol (15.1%), dihydrocarveol (9%), α-Terpineol (7.7%) Methyl eugenol (91.1%) trans-Pinocarveol (25.1%), dihydrocarveol (23.6%), myrtenol (12.3%), 1,8-cineole (11.7%)	*Raphanus sativus*, *Lepidium sativum*, *Sinapis arvensis*, *Triticum durum* and *Phalaris canariensis*	*In vitro* assays in Petri dishes. EOs were prepared in water–acetone mixture (99.5:0.5). Doses assayed: 2.5, 1.25, 0.625, 0.25, 0.125 and 0.062 μg/mL.	The EOs tested showed no effect against seed germination, but affected the radicle elongation of the five tested seeds.	[87]
*Eucalyptus tereticornis*	α-Pinene, 1, 8-cineole and β-pinene constituted more than 50% of EO composition other components in high concentrations were α-eudesmol and β-eudesmol	*Echinochloa crus-galli*	Bioassay in Petri dishes, doses tested: 0, 25, 50,100 and 250 μg/mL. Parameters evaluated: germination percentage, root and shoot length, dry weight of 7-day-old seedlings, total chlorophyll content, cellular respiration or cell survival of treated and control seedlings.	*E. terticornis* EO suppressed the growth and affected the physiology of *E. crus-galli*. Doses of 100 and 250 μg/mL affected seed germination and seedling development. A 250 μg/mL dose decreased chlorophyll content by 80% and respiratory activity by 60%. The effect on macromolecules, i.e., proteins and carbohydrates, followed a similar trend.	[79]
*Peumus boldus* *Citrus limon*	Ascaridole (31.56 ± 0.15%), p-cymene (21.58 ± 0.09%), 1,8-cineole (12.57 ± 0.13%) Limonene (59.28%), β-pinene (12.96%), γ-terpinene (10.92%)	*Portulaca oleracea*	*In vitro* germination inhibition assays in Petri dishes with filter paper, or filled with sand, or different types of soil. Soils tested: clay soil, silty clay soil, loam soil, sandy clay loam soil. Doses tested: 0.125, 0.250, 0.5 and 1 μL/mL.	*P. boldus* EO was the most effective. At the two highest doses controlled completely seed germination of *P. oleracea* in soilless culture (paper, sand and clay). At the lowest concentration applied, the EO reduced slightly seed germination in clay textural classes without effect in loam and in soilless culture. At 0.250 μL/mL *P. boldus* EO showed significant effect in clay and sand culture. The highest dose tested was effective in both soil and soilless culture. The type of soil affected *P. oleracea* germination. Probably seedling emergence declined with increasing clay content (clay and silty clay texture class), and increased with increasing sand content (sand and loam texture). *C. lemon* EO did not show any herbicidal effects at the doses tested.	[65]
*Achillea millefolium* *Anthemis cotula* *Artemisia annua* *Artemisia verlotiorum* *Bidens tripartita* *Helianthus tuberosus* *Helichrysum italicum* *Inula viscosa* *Otanthus maritimus* *Xanthium strumarium*	Artemisia ketone (25.3%), *trans*-pinocarveol (20.9%), camphor (12.9%) Not reported1,8-Cineole (23.4%), *trans*-sabinyl acetate (12.5%), artemisia ketone (12.4%), camphor (10.4%) Chrysanthenone (22.2%), 1,8-cineole (19.4%), β-pinene (16.3%) Not reported Not reported Not reported Not reported Camphor (33.6%), yomogi alcohol (18.6%), artemisia alcohol (16.3%) Borneol (30.3%), isobornyl acetate (12.2%), camphene (11.8%), limonene (11.6%)	*Amaranthus retroflexus* *Setaria viridis*	*In vitro* test in Petri dishes against seed germination of *A. retroflexus* and *S. viridis* to select the most active EOs.	*A. retroflexus* was the most sensitive weed and *S. viridis* the most resistant. *A. annua*, *A. verlotiorum* and *X. strumarium* EOs were the most active, completely inhibiting *A. retroflexus* germination at 10 µg/mL, and *S. viridis* germination at 100 µg/mL. Five EOs were selected from this experiment for their stronger herbicidal activity and were tested in in vivo conditions: *Achillea millefolium*, *Artemisia annua, Artemisia verlotiorum, Otanthus maritimus, Xanthium strumarium.*	[88]
			*In vivo* test carried out in post-emergence in containers prepared with peat substrate. The EOs tested were prepared in an aqueous solution with water using Tween 80 as emulsifier (0.1% *v*/*v*). Doses tested: 10, 100 and 1000 mg/L. EOs were applied at two different phenological stages on the weed seedlings: at cotyledons and at the third true leaf. The EOs were sprayed using a micro airbrush (AG5107, Humbrol, Kent, UK).	EOs from *A. annua* and *X. strumarium* were the most active (at the highest concentration induced the total death of all plants), but *X. strumarium* showed the highest herbicidal potential, so it was tested again to monitor the dynamics of plant damage symptoms. At the highest dose tested (1000 mg/L) plant fresh weight was reduced 20–30% 10 days after application, and chlorophyll molecules were destroyed.	
*Satureja hortensis*	Carvacrol (55.6%), γ-terpinene (31.9%)	*Amaranthus retroflexus* *Chenopodium album*	*S. hortensis* EO applied in an oil/water (O/W) nanoemulsion (NE) was tested in vitro and in vivo, in greenhouse conditions. *In vitro* germination inhibition assays in Petri dishes, concentrations tested 100, 200, 400, 800 and 1000 μL/L. A solution of 2.0% *v*/*v* Tween 80 in distilled water was used as control.	Germination inhibition increased with NE concentration. In *A. retroflexus*, the control germinated 76.6% and the maximum effect (lowest germination) was observed for 800 μL/L; the dose 1000 μL/L completely controlled *A. retroflexus* germination. In *C. album*, the germination percentages were 56.6% for the control and 16.6% for 1000 μL/L dose. A dose–response relationship was verified for the germination inhibition and for the root and shoot elongation. Germination speed (GS), was greatly dose-dependent, the lowest GS was for 800 μL/L dose, and was lower for *A. retroflexus* (0.3) than for *C. album* (0.93). The NE reduced root elongation more than shoot growth in both weeds. *S. hortensis* EO had greater effect on the seedling growth of *C. album* rather than on seed germination.	[67]
			*In vivo* assays in pots in greenhouse. The NE was applied in post emergence, in 2–4 leaves stage of the weeds by spraying with a common garden sprayer at the rate of 100 mL/m^2^. Concentrations tested: 1000, 2000, 3000, 4000 and 5000 μL/L. A solution of 1.0% *v*/*v* Tween 80 in water was used as control. Morphophysiological characteristics including fresh and dry weights (whole individual plants), leaf surface, root length and shoot length were measured and recorded 10 days after spray. Healthy plants were counted and recorded for determination of lethality percentage (LP). LP = (N/n) × 100, where “n” represents the death weeds 10 days after spray, and “N” the total number of weeds.	Weeds manifested injury symptoms 30 min after being treated. The maximum lethality was reached within 24 h after treatment application. Ten days after treatments both weeds were killed by 4000 and 5000 μL/L. The treatments with different concentrations of NE caused a significant reduction on root and shoot elongation in both weeds. The reduction was greater on roots than on shots. The growing factors length of seedlings primary root (RL), length of seedlings primary shoot (SL), leaf area (LA), fresh weight (FW) and dry weight (DW) were significantly decreased with increasing concentrations of NE. The total chlorophyll content decreased in a dose-dependent manner. NE at 1000, 2000 and 3000 μL/L concentrations provoked significant deterioration in the membrane integrity by increasing the electrolyte leakage.	
*Eucalyptus citriodora*	Citronellal (64.7%), citronellol (10.9%)	*Sinapis arvensis* *Sonchus oleraceus* *Xanthium strumarium* *Avena fatua*	*In vitro* germination inhibition experiments in Petri dishes, doses tested: 0.01, 0.02 and 0.03%.	At 0.01 and 0.02%, seed germination of all weeds tested was affected; at 0.03%, *S. arvensis* germination was completely blocked and the germination of the other weeds was strongly inhibited. Germinated seeds in treated Petri dishes with 0.02 and 0.03% doses showed a high reduction in root and shoot lengths.	[77]
			*In vivo* experiments in pots incubated in a growth chamber, doses tested: 1, 2 and 3%. 3–4 leaf stage plants were sprayed with the EO treatments, injury level was registered daily 1–6 days after treatment.	At the highest concentration (3%), 100% lethality in *S. arvensis*, *S. oleraceus* and *A. fatua* and 90% in *X. strumarium.* Total chlorophyll content was reduced depending on the EO concentration. At 1% significant electrolyte leakage was registered for all weeds except *A. fatua*. At 2–3% significant decline of membrane integrity was observed due to intense ion leakage.	
*Rosmarinus officinalis* *Satureja hortensis* *Laurus nobilis*	α-Pinene (25.85%), 1,8-cineole (9.67%), camphor (9%), camphene (7.79%) γ-Terpinene (31.98%), carvacrol (55.66%) 1,8-Cineole (40.1%), α -terpinyl acetate (14.6%), terpinene-4-ol (6.37%)	*Amaranthus retroflexus,* *Bromus tectorum* *Solanum lycopersicum*	Bioassays in Petri dishes with filter paper with the EOs prepared in solution with Tween 80, and combination of *R. officinalis* (50%) and *L. nobilis* (50%) EOs (R+L) were tested in several concentrations: 100, 200, 400, 800, 1000 and 1200 μL/L. A solution of 2.0% *v*/*v* Tween 80 in distilled water was used as control.	The tested EOs strongly inhibited the germination and seedling growth of all species, in a dose dependent manner, with *A. retroflexus* the most sensitive. At 400 μL/L, *R. officinalis* EO inhibited *A. retroflexus* germination by 91.3%; germination of *B. tectorum* and seedling growth of tomato were reduced by 56.7 and 26.7%, respectively. *R. officinalis* EO was the most active against germination of *A. retroflexus* and *S. lycopersicum* while *B. tectorum* germination was well inhibited by *S. hortensis* EO. *A. retroflexus* shoot length was inhibited by R+L EO more than by the other EOs, while the higher root growth inhibition was caused by *S. hortensis* EO. For *B. tectorum* and *S. lycopersicum*, *S. hortensis* EO showed the strongest inhibitory effect on root and shoot elongation.	[68]
*Eucalyptus citriodora* *Lavandula angustifolia* *Pinus sylvestris*	Citronellal (88.0 ± 0.8%), isopulegol (4.3 ± 1.1%) Linalool (38.7 ± 0.1%), 1,8-cineole (26.5 ± 0.0%), camphor (14.2 ± 0.1%) α-Pinene (25.6 ± 0.2%), limonene (18.5 ± 0.2%), bornyl acetate (17.9 ± 0.0%), β-pinene (15.9 ± 0.1%)	*Portulaca oleracea* *Lolium multiflorum* *Echinochloa crus-galli* *Solanum lycopersicum* *Cucumis sativus* *Nicotiana glauca*	*In vitro* assays in Petri dishes, seed germination and seedling growth were evaluated. Doses tested: 0.125–1 µL/mL.	*L. angustifolia* EO was the most active in all species tested except cucumber. *E. citriodora* and *P. sylvestris* did not reduce weed seed germination at the doses tested. *L. multiflorum* was the most sensitive weed, especially to *L. angustifolia* EO, which reduced its hypocotyl and radicle length by 87.8% and by 76.7%, at the maximum dose applied. *C. sativus* behaved as the most resistant crop, it did not show any reductions on seed germination and hypocotyl length. *L. angustifolia* could be used to control *L. multiflorum* in cucumber crops without affecting it.	[78]
*Copaifera duckei* *Copaifera reticulata* *Copaifera martii*	Leaf EO: germacrene D (23.37%), β-caryophyllene (13.92%), bicyclogermacrene (9%) Steam EO: β-caryophyllene (33.45%), germacrene D (12%), α-humulene (7.63%) Leaf EO: β-caryophyllene (20.06%), germacrene D (17.53%), δ-cadinene (6.61%) Steam EO: β-caryophyllene (24.77%), β-selinene (14.36%), germacrene D (10.61%), Leaf EO: β-caryophyllene (19.9%), germacrene D (15.82%), bicyclogermacrene (8.86%) Steam EO: α-copaene (14.41%), β-caryophyllene (9.2%), cyperene (8.25%), δ-cadinene (7.19%)	*Mimosa pudica* *Senna obtusifolia*	Steam and leave EOs were tested. *In vitro* assays in Petri dishes to test germination inhibition capacity of EOs, and their effects on root and hypocotyl development.	The inhibitory effects of both EOs against *M. pudica* and *S. obtusifolia* germination were very low (<20%). Inhibitory effects of the EOs were greater on root development than on seed germination. *M. pudica* was more sensitive than *S. obtusifolia*. Leaf oils showed strong phytotoxic activity on root and hypocotyl development (values above 42%), whereas stem oils showed greater inhibition of seed germination. *C. reticulata* was the most active EO against seed germination, and *C. martii* against root development. Against hypocotyl development, *C. reticulata* EO showed the greatest activity on *M pudica* (76% inhibition) while *C. martii* EO on *S. obtusifolia* (47.2% inhibition).	[89]
*Thymbra capitata* *Mentha* × *piperita* *Santolina chamaecyparissus* *Eucalyptus camaldulensis*	Carvacrol (72.30%), *p*-cymene (8.93%), γ-terpinene (7.77%) Menthol (51.81%), menthone (20.52%) 1,8-Cineole (17.50%), viridiflorol (13.56%), germacrene-D (12.60%) Spathulenol (31.29%), *p*-cymene (20.36%), cryptone (17%)	*Erigeron bonariensis*	*In vivo* assays in pots, in greenhouse conditions, in pre- and post-emergence. EOs emulsified with water using Fitoil (1 µL/mL). In pre-emergence EOs were applied by watering, in post-emergence EOs were applied by watering and by spraying. Doses tested 2, 4 and 8 µL/mL.	All the EOs inhibited *E. bonariensis* germination, with the most effective being *T. capitata*, followed by *E. camaldulensis* and *S. chamaecyparissus*. In post-emergence assays, *T. capitata* EO was the most active in both modes of application but acted more rapidly when sprayed. The second most active EO was *E. camaldulensis* applied by watering and also *M. piperita* EO applied by spraying showed a similar effectiveness.	[69]
*Thymbra capitata*	Carvacrol (72.30–77.13%)	*Erigeron canadensis* *Sonchus oleraceus* *Chenopodium album* *Setaria verticillata* *Avena fatua* *Solanum nigrum* *Amaranthus retroflexus* *Portulaca oleracea* *Echinochloa crus*-*galli*	*In vitro* assays in Petri dishes, EO applied directly to the filter paper. Doses tested: 0.125, 0.5, 0.5, 1 and 2 µL/mL.	The EO controlled completely the germination and seedling development of *E. canadensis*, *S. oleraceus* and *C. album* (more sensitive species) at 0.125 μL/mL, of *S. verticillata*, *A. fatua* and *S. nigrum* at 0.5 μL/mL, of *A. retroflexus* at 1 μL/mL and of *P. oleracea* and *E. crus-galli* (more resistant species) at 2 μL/mL.	[72]
*Thymbra capitata*	Carvacrol (72.30–77.13%)		Tests against the seedbank of a citrus orchard, in trays, in greenhouse conditions, EO emulsified with water using Fitoil (1 μL/mL) applied in pre- and post-emergence, doses tested: 1, 2 and 4 µL/mL.	Good results with the dose 4 µL/mL.	
*Thymbra capitata*	Carvacrol (72.30–77.13%)	*Erigeron bonariensis* *Portulaca oleracea* *Avena fatua* *Echinochloa crus-galli* *Erigeron bonariensis*	*In vivo* assays in greenhouse conditions, EO applied in post-emergence, when the plants had 2–3 true leaves (stage 12–13 BBCH scale) for the monocotyledons, and 3–4 true leaves (stage 13–14 BBCH scale) for the dicotyledons, by watering or by spraying, emulsified with water using Fitoil (0.5 μL/mL). Doses tested: 4, 8 and 12 μL/mL for all species, except *E. bonariensis*, on these species the doses tested were 2, 4 and 8 μL/mL.	*E. crus-galli* was the most resistant species. Comparing results with the EO applied by irrigation and by spraying, it was concluded that at the same doses it was more effective on monocotyledons applied by irrigation and on dicotyledons by spraying. The EO applied by spraying controlled completely *P. oleracea* at all doses applied, *E. bonariensis* at 2 and 8 µL/mL and *A. fatua* at the highest dose applied. On *E. crus-galli* was not effective when applied by spraying. The EO applied by irrigation at the highest dose totally controlled *A. fatua*, while *P. oleracea* and *E. bonariensis* were controlled in 90% and *E. crus-galli* 50%.	
*Thymbra capitata* *Mentha* × *piperita* *Santolina chamaecyparissus*	Carvacrol (72.30%), p-cymene (8.93%), γ-terpinene (7.77%) Menthol (51.81%), menthone (20.52%) 1,8-Cineole (17.50%), viridiflorol (13.56%), germacrene-D (12.60%)	*Amaranthus retroflexus* *Portulaca oleracea* *Avena fatua* *Echinochloa crus-galli*	*In vivo* assays in pots in greenhouse conditions. EOs applied in post-emergence. EOs applied by watering and by spraying, emulsified with water using Fitoil (0.5 μL/mL). Doses tested: 4, 8 and 12 µL/mL *T. capitata* EO; 12, 16 and 20 µL/mL *M. piperita* EO; 12, 16 and 20 µL/mL *S. chamaecyparissus* EO.	*T. capitata* EO was the most effective, killing all plants of all weed species at the highest dose applied, except *P. oleracea*, which was eliminated in 90%. *M. piperita* EO also showed good herbicidal potential, especially against *A. retroflexus* and *A. fatua*, which were the most sensitive weeds to all EOs tested, while *P. oleracea* and *E. crus-galli* were the most resistant. *S. chamaecyparissus* was the least active EO; it controlled some plants but did not eliminate completely any species.	[71]
17 Eucalyptus EOs tested *Eucalyptus nutans* *E. resinifera* *E. carnei* *E. amplifolia* *E. angulosa* *E. fastigata* *E. selachiana* *E. albida* *E. grandis* *E. planchoniana* *E. exserta* *E. saligna* × *E. exserta* No. 9 (the optimizing of 9 lines of *E. saligna* × *E. exserta*) *E. urophylla* No. 4 *E. urophylla* No. 16 *E. urophylla* × *E. camaldulensis* No. 3 *E. grandis* × *E. urophylla* No. 5 E. *grandis* × E. *urophylla* No. 9	1,8-Cineole (23.36%), α-pinene (14.65%), viridiflorol (7.58%), *trans*-pinocarveol (6.67%) 1,8-Cineole (37.19%), α-pinene (13.82%), *trans*-pinocarveol (10.55%), borneol (7.40%) 1,8-Cineole (19.21%), α-pinene (34.19%), *trans*-pinocarveol (8.27%) 1,8-Cineole (17.57%), α-terpinyl acetate (11.26%), α-pinene (7.64%), globulol (6.77%), *trans*-pinocarveol (6.77%), aromadendrene (6.65%) α-Pinene (17.61%), 1,8-cineole (16.58%), globulol (12.36%), *trans*-pinocarveol (9.77%) β-Eudesmol (31.90%), γ-eudesmol (17.32%), 1,8-cineole (12.17%), α-pinene (9.22%) α-Pinene (13.25%), 1,8-cineole (11.62%), globulol (7.54%), terpinolene (7.54%), β-eudesmol (7.53%) α-Pinene (22.27%), 1,8-cineole (11.49%), isobutyl isobutyrate (11.19%) 2,2,5,5-Tetramethyl-4-(2-hydroxy-2-methylbutylidene) cyclopenta-1,3-dione (13.17%), flavesone (9.29%), *iso*-leptospermone (8.11%) β-Eudesmol (20.02%), α-pinene (11.63%), 1,8-cineole (11.02%) 1,8-Cineole (46.31%), p-cymene (20.41%), α-pinene (9.75%) 1,8-Cineole (55.31%), (Z)-β-ocimene (19.23%), α-terpinyl acetate (15.04%)1,8-Cineole (64.09%), α-pinene (11.40%) 1,8-Cineole (62.74%), α-pinene (22.64%) 1,8-Cineole (43.89%), α-pinene (16.28%), limonene (26.61%) 1,8-Cineole (64.34%), α-pinene (12.89%) 1,8-Cineole (63.69%), α-pinene (13.12%)	*Lolium rigidum*	Germination and growth inhibition test in Petri dishes. EOs were added at doses of 0, 0.25, 0.5, 2.5, 5.0 and 7.5 μL in each Petri, previously filled with 5 mL of water.	Effects depended on the *Eucalyptus* species EO tested. At 5.0 μL/dish and 7.5 μL/dish, 100% germination inhibition by *E. grandis*, *E. resinifera* and *E. angulosa*. At 5.0 μL/dish and 7.5 μL/dish, *E. ampflifolia* and *E. carnei* inhibited the shoot growth by 96.6 and 93.2%. At 7.5 μL/dish, *E. carnei* and *E. amplifolia* inhibited the root growth by 99.3 and 93.0%.	[74]
			Germination and growth inhibition test in Petri dishes. Pure compounds 1,8-cineole, α-pinene, α-terpineol, trans-pinocarveol, γ-terpinene and terpinolene were tested at the doses of 0, 0.25, 0.5, 2.5, 5.0 and 7.5 μL) in each Petri, previously filled with 5 mL of water.	At 2.5 μL/dish, α-terpineol and *trans*-pinocarveol reduced the germination by 98.9 and 96.4%, while the others less than 14.8%.	
			Pot experiment was conducted with α-pinene (weak herbicidal activity) and α-terpineol and *trans*-pinocarveol (strong herbicidal activity). Doses tested: 0, 0.0625, 0.125, 0.25, 0.8 and 1.0 mL/g soil.	At 0.5 μL/g and 1.0 μL/g, *trans*-pinocarveol totally controlled *L. rigidum* germination, α-terpineol inhibited the germination by 49.0 and 89.7%, respectively, and α-pinene reduced germination by 43.9 and 56.0%, respectively. The IC_50_ value was 0.16 μL/g for α-terpineol and 0.19 μL/g for *trans*-pinocarveol. The IC_50_ of control herbicide pendimethalin was 0.017 μL/g	

**Table 2:** Effects and metabolic targets of phytotoxic terpenoids on different species. Manuscripts focused on the screening of the phytotoxic potential of pure molecules, that reported only information of their effects on germination or shoot and root growth parameters, were not included in the table.

Molecule	Species	Effects	Bibliography
Monoterpenoids			
α -Pinne	*Zea mays*	Altered the mitochondrial respiration by inhibiting the electron transport chain	[132]
	*Cassia occidentalis*	Enhanced solute leakage in roots, increased H_2_O_2_ content and lipid peroxidation inducing oxidative stress; stimulated the production of the osmoprotectant proline	
ß-Pinene	*Oryza sativa*	Reduced chlorophyll content, enzymatic activity of proteases, cell respiration and α- and ß-amylases; stimulated the activity of polyphenol oxidases and peroxidases	[133]
Citral	*Triticum aestivum*	Reduced cell division, disrupted mitotic microtubules and cell plates, and inhibited cell elongation by damaging cortical microtubules	[108]
	*Arabidopsis thaliana*	Altered auxin content, cell division and ultrastructure inducing cell wall thickening, damages to mitochondria, chromatin fragmentation and reduction in intercellular communication (plasmodesmata alteration)	[109]
	*Arabidopsis thaliana*	Altered plant metabolism, induced oxidative stress and damages to the photosynthetic machinery reducing its efficiency	[121]
	*Arabidopsis thaliana*	Altered plant water status and plant fitness (plants were unable to produce siliques and seeds), increased anthocyanin content (osmoprotectant)	[122]
	*Arabidopsis thaliana*	Inhibition of DNA transcription by competing with the strand-binding proteins	[112]
	*Allium cepa*	Strongly inhibited metaphase, anaphase and telophase and induced cellular aberration (stickiness, binucleated cell, disturbed anaphase–telophase, chromosomal ring, c-mitosis, chromosomal fragmentation, mitotic bridge, vagrant, polyploidy, laggard)	[119]
Citronellal	*Cassia occidentalis*	Reduction of chlorophyll content and cell respiration	[134]
Eugenol	*Cassia occidentalis* and *Bidens pilosa*	Reduced chlorophyll content, photosynthetic efficiency and cellular respiration; altered the mitotic activity disorganizing the microtubule organization and altering the biosynthesis of cell wall	[135]
Limonene	*Zea mays*	Stimulated the basal respiration in isolated mitochondria and inhibited the coupled respiration, triggering loosening of respiratory control	[95]
	*Allium cepa*	Induced chromosomal and nuclear aberrations (sticky chromosomes, polynucleated cells, among others)	[119]
Menthol	*Arabidopsis thaliana*	Induced an alteration of plant water status, enhancing excessive transpiration. In fact, it induced protoplasts swelling, blocked stomatal closure altering the cytoskeleton organization (it has a pivotal role in stomatal movements); downregulated the expression of PEPCase (enzyme with an important role during stomatal opening)	[124]
	*Cucumis sativus*	Increased the concentration of cytosolic free calcium ions (Ca^2+^)	[136]
	*Zea mays*	Induced the production of malondialdehyde, peroxides and conjugated dienes inducing, as a consequence, oxidative stress	[137]
Pulegone	*Cucumis sativus*	Acted as an uncoupler agent of mitochondrial respiration	[136,138]
Thymol	*Arabidopsis thaliana*	Altered the plant water status, induced oxidative stress and physical damages to the photosynthetic machinery; plants responded by accumulating metabolites (amino acids, sugars, anthocyanins, among others) and protein involved in oxidative stress defense	[127]
S-Carvone	*Solanum tuberosum*	Reduced the activity of the 3-hydroxy-3-methylglutaryl coenzyme A reductase without affecting its mRNA level	[138]
Camphor	*Allium cepa*	Inhibited mitosis and cell respiration	[103,139]
	*Arabidopsis thaliana*	Altered the cuticular waxes structure and enhance leaf transpiration causing water losses and cell dehydration	[123]
	*Arabidopsis thaliana*	Disrupted actin filaments and microtubules affecting cell division	[124]
1,8-Cineole	*Avena fatua*	Inhibited mitochondrial respiration in isolated organelles	[139]
	*Allium cepa*	Inhibited mitosis in all its stages	[81]
	*Allium cepa*	Induced swelling of the root tips, phenomenon known to be caused by an alteration of the cortical microtubules	[103]
1,4-Cineole	*Allium cepa*	Inhibited the prophase of mitosis and induced malformations in the shoot, which was characterized by corkscrew-shape (handedness).	[81]
	*Echinochloa crus-galli, Cassia obtusifolia*	Inhibited the photosynthetic efficiency causing a reduction of plant growth and development	[81]
	*Ageratum conyzoides*	Disrupted the microtubule organization and altered the biosynthesis of cell wall; in addition, it induced a decrease in chlorophyll content and cell respiration reducing the photosynthetic activity	[82]
**Sesquiterpenes**			
Farnesene	*Arabidopsis thaliana*	Altered the auxin/ethylene balance inducing an alteration of the microtubule organization and density, as a consequence induced a loss of the gravitropism due to an anisotropic growth of the primary root (phenomenon known as left-handedness); induced cell ultrastructure alterations (swollen cell walls, broken mitochondria, polynucleated cells etc.)	[114]
	*Arabidopsis thaliana*	Affected auxin transport (inhibited all PIN proteins) and distribution causing the alteration of cell shape in the root meristem, and consequently the left-handedness phenotype.	[113]
*trans*-Caryophyllene	*Arabidopsis thaliana*	Induced alterations to plant water status followed by oxidative stress and physical damages to the photosynthetic machinery; moreover, the rosettes of plants treated were characterized by a cork-screw shape indicating microtubular alterations	[125]
Nerolidol	*Arabidopsis thaliana*	Induced alterations in root morphology mediated by an auxin unbalance and ROS accumulation; metabolomic analysis pointed out changes in sugar, amino acid and carboxylic acid profiles causing a strong impact on starch and sucrose metabolism, alanine, aspartate and glutamate metabolism, and glycine, serine and threonine metabolism.	[116]
**sesquiterpene endoperoxide**			
Artemisinin	*Vigna radiata*	Inhibited the synthesis of the enzyme peroxidase	[140]
	*Arabidopsis thaliana*	Altered the root gravitropic responses reducing the number of starch grain in the columella cells and altering auxin lateral distribution (inhibition of the PIN2 carrier)	[118]
	*Lactuca sativa*	Induced reactive oxygen species accumulation, lipid peroxidation and cell death	[117]
	*Beta palonga* and *Oryza sativa*	Its biological activity was mainly due to the products of artemisinin metabolization; it induced a strong inhibition of the photosynthetic electron transport affecting, as target site, the secondary quinone moiety of photosystem II complex (Q_B_)	[128]
	*Arabidopsis thaliana*	Altered the photosynthetic machinery reducing the photosynthetic efficiency, PSII yield and the electron transport rate	[129]
	*Lemna minor*	Inhibited cell respiration	[141]
	*Lactuca sativa*	Stimulates oxygen uptake in root tips, altered all the mitotic phases inducing mitotic aberrations	[142]
	*Lemna minor*	Induced the release of proteins in the culture medium as a consequence of plasma membrane alterations	[143]
**sesquiterpene lactone**			
dehydrozaluzanin C	*Lactuca sativa*	Induced electrolyte leakage as a consequence of the separation of the plasma membrane from the cell wall	[144]
**Diterpenes**			
Podolactones (nagilactone C, podolactone A and podolactone E)	Several species (lettuce, barley, *Arabidopsis, Lolium temulentum*	Inhibited the biosynthesis of chlorophyll, induced the swelling of root tip (cortical microtubule alteration) and inhibited the hormone-induced growth	[145]
Podolactone A	*Pisum sativum*	Suppressed auxin- induced growth and proton efflux without affecting ATP levels	[146]
Podolactone E	*Hordeum vulgare*	Inhibited the biosynthesis of δ-aminolevulinic acid and chlorophyll	[147]
**Triterpenes and derivatives**			
Holacanthone	*Allium cepa*	Inhibited all stages of mitosis	[148]
Chaparrinone	*Allium cepa*	Inhibited all mitotic stages excluded profase	[148]
Glaucarubolone	*Allium cepa*	Inhibited all mitotic stages excluded profase	[148]
Digitonin (saponin)	*Catharanthus roseus*	Induced callose biosynthesis and Ca^2+^ uptake involving transport proteins controlled by protein phosphorylation/dephosphorylation	[149]
Betulin	*Allium cepa*	Altered the formation of the spindle microtubular organization centers, causing the development of multiple spindle poles and a chromosome asymmetrical convergence	[150]
